# Inflammatory response after stroke—A clinical observation study

**DOI:** 10.1186/s12883-025-04244-y

**Published:** 2025-05-30

**Authors:** Björn Röyter, Nellie Andréasson, Bianka Forgo, Jan Källman, Jakob O. Ström

**Affiliations:** 1 Department of Obstetrics and Gynecology, Faculty of Medicine and Health, Örebro University, SE-701 82 Örebro, Sweden; 2 Department of Geriatrics, Faculty of Medicine and Health, Örebro University, SE-701 82 Örebro, Sweden; 3 Department of Neuroradiology, Karolinska University Hospital, Stockholm, Sweden SE-171 76; 4 Department of Clinical Neuroscience, Karolinska Institutet, Karolinska University Hospital, Stockholm, Sweden SE-171 76; 5 Faculty of Medicine and Health, Örebro University, SE-701 82 Örebro, Sweden; 6 Department of Infectious Diseases, Faculty of Medicine and Health, Örebro University, SE-701 82 Örebro, Sweden; 7 Department of Neurology, Faculty of Medicine and Health, Örebro University, SE-701 82 Örebro, Sweden

**Keywords:** Stroke, Infection, WBC, CRP, Temperature

## Abstract

**Background:**

Body temperature and peripheral blood inflammatory markers are often elevated in acute stroke. Whether the increase in inflammatory markers is caused by the stroke itself or is attributable to a complication, is incompletely understood. This uncertainty may hamper the diagnosis and treatment of infections. We aimed to describe the dynamics of inflammatory parameters in a cohort of stroke patients free from complications.

**Methods:**

Acute stroke patients were prospectively included within 48 h of symptom onset and monitored through daily questions of symptoms and clinical examinations to detect complications. Inflammatory parameters in blood and body temperature were measured daily for up to ten days and the 97.5th percentile calculated. Values were compared with paired t-test to measurements at a 90-day follow up.

**Results:**

70 stroke patients were included. 51 of them were considered complication-free and sampled for a total of 282 days. Body temperature, CRP and WBC were all significantly elevated the first days after stroke, compared to 90-days post stroke. Mean body temperature was highest at 24-48h at 37.1°C, mean WBC was highest at 0-24h at 8.1 × 10^9/L, compared to 36.7°C and 6.0 × 10^9/L at the 90-day follow-up (*p*-values < 0.01). Median CRP peaked at 7.0 mg/L 120–144 h after stroke, compared to 0.9 mg/L at follow-up (*p*-value < 0.01).

**Conclusions:**

Acute stroke may cause mildly elevated levels of CRP, WBC and body temperature. Except for WBC during the first 24h, higher levels (such as CRP > 50mg/L, WBC > 11 × 10^9/L or body temp > 38°C) are very uncommon (< 2.5%) and are likely to reflect a complication.

**Supplementary Information:**

The online version contains supplementary material available at 10.1186/s12883-025-04244-y.

## Background

Stroke is the second leading cause of death globally [1]. Preventive treatment of risk factors and revascularization treatment during the first hours of cerebral infarction have taken leaps in the last decades. However, complications in the subacute phase of stroke, such as infections, remain an incompletely understood area. Infections may affect about 30% of stroke patients, with the two most common being pneumonia and urinary tract infections (UTI) [2]. Pneumonia is especially concerning and has been estimated to cause almost a third of all stroke deaths [3].

There are several reasons why stroke patients are vulnerable to infection. Many become bedbound, suffer from dysphagia, and receive urinary catheters or intravenous lines, prone to introduce bacteria [4]. There is also mounting evidence that stroke and other types of brain injuries elicit immunosuppression [5, 6].

In the assessment of a stroke patient with suspected infection, clinicians face a complex differential diagnostic challenge since stroke patients are prone to develop a number of febrile complications. These include deep vein thrombosis (DVT), pneumonia, UTI, aseptic aspiration pneumonitis, and pressure ulcers. These may present with fever or elevated biomarkers of infection. In addition, there is a common perception among neurologists that the stroke itself may cause endogenous, non-infectious fever (analogous to fever after myocardial infarction [7]).

Some studies have shown an increase in commonly used markers of infection, such as C-reactive protein (CRP) and white blood cell count (WBC) [8], however, other results have refuted this [9]. These discrepancies could be attributable to differences in stroke severity or measurement time-points, but one especially challenging confounder in studies of this type is undiagnosed infections. Since infections are very common and patients have a plethora of symptoms from the stroke itself, they are easily under-diagnosed. The majority of studies on this topic are conducted using a retrospective method for detecting infections, through chart review. Only one has a prospective method [9]. Altogether, there is no robust evidence on whether infection-free stroke patients are expected to have elevated inflammatory parameters or not. Increased knowledge on the natural dynamics of inflammatory markers is imperative since it may justify other reference intervals for acute stroke patients and may guide the workup for complications.

The primary aim of this study was to determine a range within which values of body temperature, CRP and WBC are expected in complication-free stroke patients. A secondary aim was to investigate in what way the stroke itself seemed to contribute to the elevations of body temperature, CRP and WBC.

## Methods

This was a prospective observational cohort study of stroke patients at Örebro University Hospital, Sweden, following patients up to 10 days during hospitalization after stroke and at a 90-day follow-up.

### Subjects

Our study consecutively included patients ≥ 18 years of age with acute ischemic stroke or spontaneous intracerebral hemorrhage admitted to the stroke units between January 2019 and October 2022, with onset of stroke symptoms within 48 h of study admission. Patients with complications known to cause fever (definitions below) were excluded from analysis. Stroke diagnosis was based on clinical symptoms and radiological findings. Patients with a negative magnetic resonance imaging (MRI) of the brain or a clinical transient ischemic attack (TIA) diagnosis were consequently excluded. As inflammatory parameters were of main interest and repeated blood sampling was conducted in the present study, patients presenting with hemoglobin < 90 g/L or a preexisting active inflammatory illness (see supplement for details) were excluded. Since a few of the exclusion criteria were based on study examinations, cases of confirmed non-eligibility after initial consent and data collection were expected. Patients were not re-included in case of a second stroke event.

Patients were recruited consecutively, except for time periods when inclusion had to be suspended, most notably due to the COVID-19 pandemic. Since fever has previously been related to stroke severity, the original intent of the cohort was to recruit a skewed sample of stroke patients with a higher proportion of severe stroke. Therefore, from September 2020 to December 2021 only patients with National Institutes of Health Stroke Scale (NIHSS) > 5 were included. Even though this is more than a year, during long periods of this time, the study was suspended and only 9 patients were recruited using these criteria. This method was deemed unfeasible and so during the subsequent study period, patients were prospectively recruited regardless of stroke severity. Since the project aims to address other scientific questions using the same cohort of patients, study size was decided considering several aims. A study size of 70 patients, with a 30% complication rate yields approximately 50 complication free patients. This results in a standard error of the mean of 14% of the standard deviation of the measurement of interest, which was considered sufficient for the main aims.

### Identification of complications

Patients were questioned and examined for symptoms of complications such as pneumonia, UTI and DVT every day based on the study protocol (see supplemental material). Patients who met our criteria for a complication were excluded from analysis. Complications were defined as symptoms of bacterial respiratory tract infection, upper urinary tract infection, gastroenteritis, venous thrombosis, infected pressure ulcer or wound with a duration of at least 2 days and in combination with some objective measurement, such as decreased saturation for respiratory tract infection or leukocyturia for urinary tract infection. Details regarding criteria can be found in the supplemental material.

No bacterial cultures or antigen tests were performed as part of the study but were sometimes performed by the treating physician. Since bacterial infections are common even with negative swabs, we decided to not include this as a mandatory criterion. This is in line with the Pneumonia in Stroke Consensus Group recommendation, where swabs are not mandatory [10].

Blood tests and any abnormal examination results were also forwarded to the treating physician for further investigation. Patients who, by the treating physician, were diagnosed with and/or received antibiotic treatment for a complication were also excluded from analysis.

### Clinical and demographic assessment

Body temperature was measured every morning for up to 10 days using a rectal thermometer. Following standard proceedings nurses could, based on their own assessment, administer antipyretics if patients were (sub)febrile or in pain but patients will have had a night-long antipyretic (including aspirin) free interval before measurement. NIHSS was measured at inclusion in the study by trained research nurses. For the majority of patients, NIHSS was also measured at admission to hospital by the on-call physician. These are referred to as NIHSS at inclusion and at admission, respectively. Since inclusion in the study sometimes occurred after thrombolysis and/or thrombectomy, we believed NIHSS at admission would be the most representative of the initial insult but unfortunately, we expected some missing data. Smoking status was collected at inclusion from patients or next of kin. Mortality, reperfusion treatment, length of stay, medications and comorbidities were collected from electronic patient registries.

### Blood sampling

Blood samples were drawn and analyzed within one hour at inclusion and daily for up to 7 days. WBC was also measured at admission. High sensitivity CRP (hsCRP) measurement was performed on a Siemens ADVIA 1800 Chemistry System according to manufacturer instructions. A 6-point calibration curve and controls were also assayed to confirm an accurate measurement according to SS-EN ISO/IEC 15 189, STAFS 2011:33 and STAFS 2010:10 (SWEDAC). Leucocyte particle concentration (WBC) analysis was performed on a Sysmex XN 9000 system with a flow cytometer method according to the manufacturer’s instructions. Controls were also assayed to confirm an accurate measurement according to SS-EN ISO/IEC 15 189, STAFS 2011:33 and STAFS 2010:10 (SWEDAC).

### Radiological image acquisition and analysis

Non-enhanced computed tomography (CT) of the head was performed at hospital admission on a Siemens Somatom Definition AS + or a Siemens Definition Edge 128-slice scanner (Siemens, Erlangen, Germany). The following acquisition parameters were used: tube voltage 120 kVp, collimation 128 × 6 mm, pitch factor 0.55, tube current as per automatic exposure control. J37 s or HC40 s convolution kernel was applied. Contiguous image sections were reconstructed with 1-mm slice thickness and 512 × 512 matrix.

Patients with radiologically or clinically diagnosed ischemic infarction underwent magnetic resonance imaging (MRI) three days after admission on a Philips Achieva dStream 1.5 Tesla scanner (Philips, Best, The Netherlands). The protocol included 2-dimensional T2-weighted axial and diffusion-weighted images (DWI). T2-weighted images were acquired using the following parameters: repetition time (TR) 4000 ms, echo time (TE) 110 ms, slice thickness 3 mm, in-plane resolution 0.4 × 0.4 mm. DWI was obtained with b-value 1000 s/mm^2^, TR 3704 ms, TE 92 ms, slice thickness 5 mm and 1.3 × 1.3 mm in-plane resolution.

Volumes of brain parenchymal hemorrhage and ischemic infarct were assessed on imaging. All measurements were made by the same board-certified radiologist who is also a neuroradiology trainee. Hemorrhage volumes were calculated on thin-section non-enhanced CT and ischemic infarct volumes were quantified using DWI. Apparent diffusion coefficient maps and T2-weighted images were used to facilitate the delineation of acute infarcts. All volumes were obtained using MRIcron [11] with semi-automatic segmentation and manual correction if needed.

Infarcts were mapped to 20 different regions, 8 bilateral: the frontal, parietal, temporal and occipital lobes, the insula, thalamus, corona radiata and cerebellum, and two medial, the brainstem and basal ganglia. To reduce model complexity for the multivariate regression, the bilateral areas were merged, and each patient was classified according to the most affected area.

### Statistical analysis

R version 4.3.2 with the tidyverse package was used for statistical analysis. Distribution was assessed with histograms and Shapiro–Wilk tests. Mean +—1.96 SD was used to illustrate the range in which 95% of patients should reside, a range commonly used for reference values. CRP was treated as log-normally distributed and paired T-tests, as well as estimation of suggested reference values (mean ± 1.96 SD) were calculated on log-transformed data. Data was presented reverse-transformed with median. Normally distributed data including body temperature and WBC were presented with mean. Paired t-tests were used to analyze elevations compared to 90-day follow-up visits. Pairwise (instead of groupwise) comparisons with measurements at 90-days ensures differences are not a result of a biased loss to follow-up. Since missing data during the first ten days was most commonly caused by patients being discharged, the unadjusted sample is representative of patients the clinician meets in a hospital setting and is presented as is. Spearman’s Rank Correlation Coefficient (Spearman’s Rho) was used to analyze the correlation between non-parametric or non-normally distributed data such as when body temperature, CRP and WBC were correlated to NIHSS and hemorrhagic and infarction size. A *p*-value < 0.05 was considered statistically significant. No corrections of the p-values were made for multiple comparisons.

A multiple regression analysis was performed in two steps. The day with the most difference (in terms of CRP, WBC and body temperature, respectively) compared to the re-visit was chosen for this analysis, with a pre-requisite of having at least 40 patient measurements that day. First, all comorbidities, sex, age, stroke type (infarct/hemorrhage), Trial of Org 10,172 in Acute Stroke Treatment(TOAST)-classification [12], infarct size and location on MRI, and NIHSS on admission were tested for univariate association with CRP, WBC and body temperature, respectively, using ANOVA, Students T-test or Spearman’s Rank Correlation as appropriate. Secondly, all variables with a p-value under 0.1 in the univariate tests were included in the respective multiple linear regression models, one for CRP, one for WBC and one for body temperature.

## Results

**Fig. 1:**
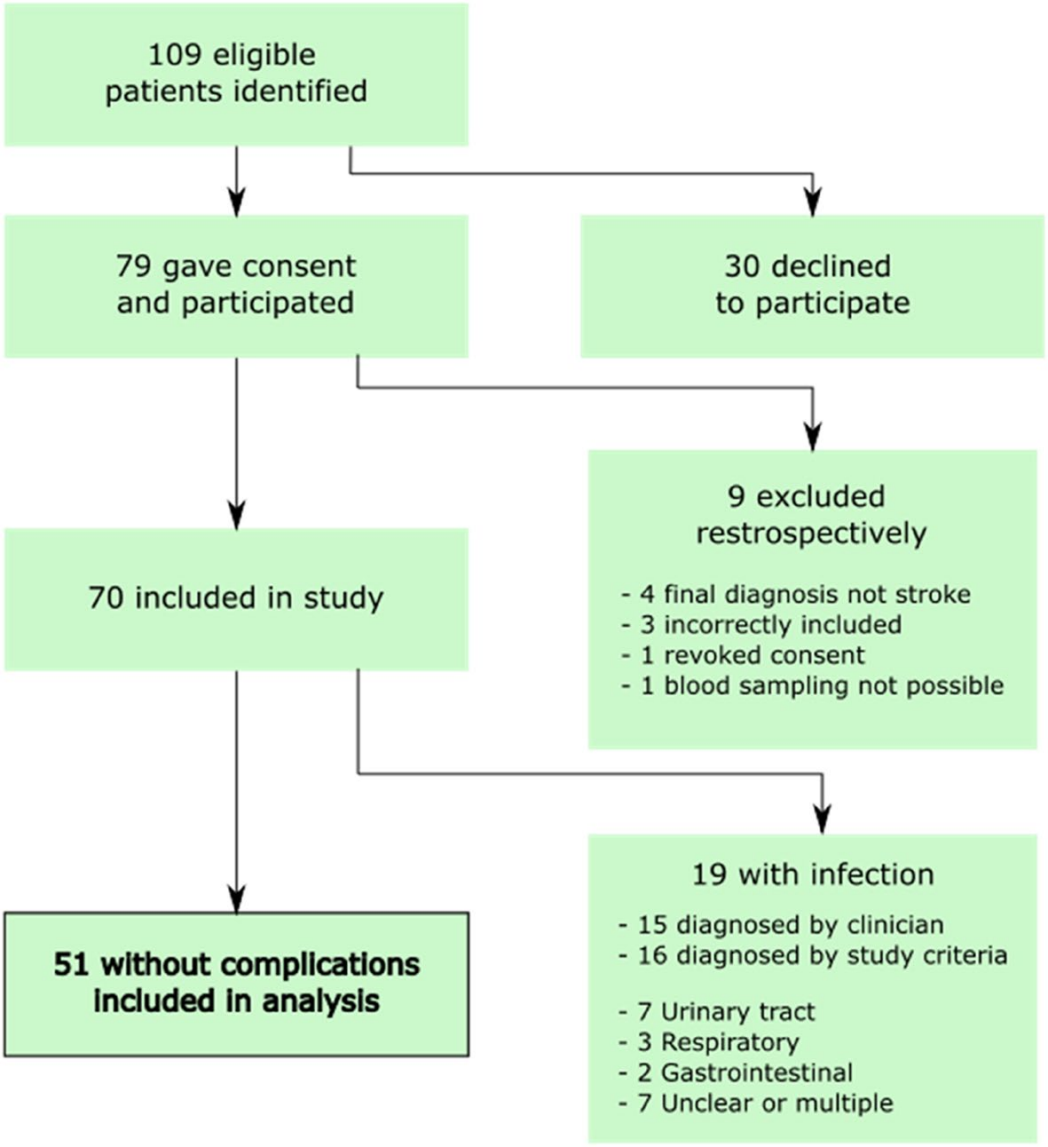
Flowchart of included and excluded patients. For patients diagnosed with infection, there was a significant overlap between the clinician’s diagnosis and diagnosis by study criteria, therefore, the total (19) is less than the sum of the parts

109 patients were identified as eligible of which 79 gave their consent and participated in study sampling and examinations. 9 patients were retrospectively excluded for not meeting pre-determined criteria, such as no stroke visible on MRI or complete resolution of symptoms within 24 h i.e. TIA. Complications were identified in 19 patients, leaving 51 patients included in the analysis (Fig. 1). Of the 19 patients, 4 had their complications detected only by the study criteria primarily based on the study questionnaire and study examinations. 3 patients had their complications only detected by the treating physician based on all information at his or her disposal. The remaining 12 patients had their complications detected by both the clinician and the study criteria. Descriptive data on patients removed from analysis is presented in the supplementary material.

One patient was taking hydroxycarbamide, a treatment for myeloproliferative diseases. Since this was not an a priori defined exclusion criteria, the patient was included in the study. The WBC-results were however removed from the analysis. Patient characteristics are presented in Table 1 and infarct subtypes and locations are presented in Table 2.

**Table 1:** Population characteristics. NIHSS: National Institutes of Health Stroke Scale

	Total (*n* = 51)	Ischemic (*n* = 44)	Hemorrhagic (*n* = 7)
Mean Age	75.0	75.5	72.0
Women	24 (47%)	22 (50%)	2 (29%)
Median NIHSS at admission	5 (*n* = 36)	5 (*n* = 31)	7 (*n* = 5)
Median NIHSS at inclusion	4	4	7
Underwent MRI examination		89%	
Median infarction volume		3.4 ml	
Median hemorrhage volume			5.9 ml
Hypertension	69%	66%	86%
Hypercholesterolemia	22%	23%	14%
Atrial fibrillation	29%	32%	14%
Diabetes	14%	9%	43%
Ischemic heart disease	14%	11%	29%
Obstructive lung disease	10%	11%	0%
Current smoker	17%	18%	0%
Former smoker	43%	39%	43%
Attended follow-up visit	63%	66%	43%
1-year mortality	12%	9%	29%
Length of stay (median days)	5	5	9
Beta-blocker treatment	53%	48%	86%
Acetaminophen treatment (regularly)	4%	5%	0%
Thrombectomy	10%	11%	
Thrombolysis	16%	19%

**Table 2:** Infarct subtypes, TOAST: Trial of ORG 10172 in Acute Stroke Treatment

**TOAST subtype (*n*** **= 44)**		
Cardioembolic	27%	
Lacunar	11%	
Large artery atherosclerosis	4%	
Unknown	52%	
**Regions affected by Infarct (n = 39)**	Right (or medial)	Left
Basal ganglia	21%	
Brain stem	10%	
Cerebellum	5%	10%
Corona radiata	21%	15%
Frontal lobe	31%	28%
Insula	10%	10%
Occipital lobe	10%	18%
Parietal lobe	28%	16%
Temporal lobe	10%	13%
Thalamus	3%	0%

### Body temperature

**Fig. 2:**
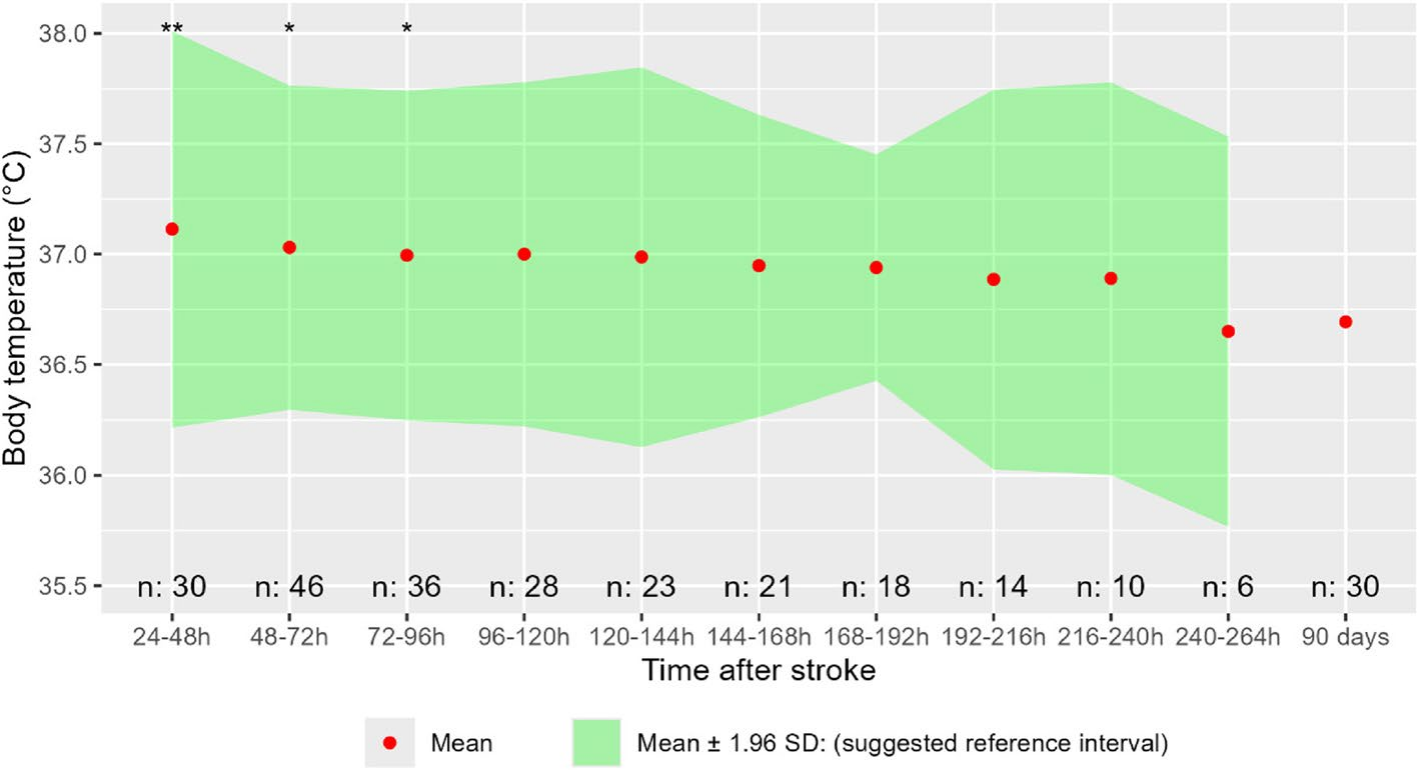
Body temperature in patients without complications. Difference tested day for day compared to 90-day follow-up using paired T-test, *p*-value: * = < 0,05, ** = < 0,01. “*n*:” is the number of observations from the respective days

Body temperature was slightly elevated during hospitalization as compared to 90-day follow up, with a highest mean temperature of 37.1 °C 24–48 h after stroke, compared to 36.7 °C at the 90-day follow-up (Fig. 2). 24–48 h after stroke, the upper limit of a computed reference interval (mean + 1.96SD) was 38.0 °C. This means that only 2.5% of stroke patients without (having or developing) an overt complication can be expected to have a morning temperature of 38.0 °C or above at 24-48 h after stroke. All other measured days, mean + 1.96SD was lower than 38.0 °C. Two patients (4%) were on a regular dose of acetaminophen.

No significant correlation was found at any day between body temperature and NIHSS at admission or inclusion, infarct size or size of hemorrhagic stroke. There was a slight and statistically insignificant positive correlation to several of these parameters at some of the days. (Figs. 1–4 in supplemental material).

As described above, the multiple regression analysis was preceded by a univariate test to exclude factors with low explanatory value. From the univariate tests, TOAST-classification, atrial fibrillation and infarct location were all associated with temperature at 48–72 h after stroke with p-values < 0.10 (0.002, 0.046 and 0.015 respectively). In the multiple regression model using TOAST-classification, atrial fibrillation and infarct location, only the TOAST-classification remained significant (*p* = 0.0041). A Tukey post-hoc test of TOAST-classification revealed that cardioembolic strokes had significantly higher temperatures compared to lacunar strokes and strokes of unknown cause.

### CRP

**Fig. 3:**
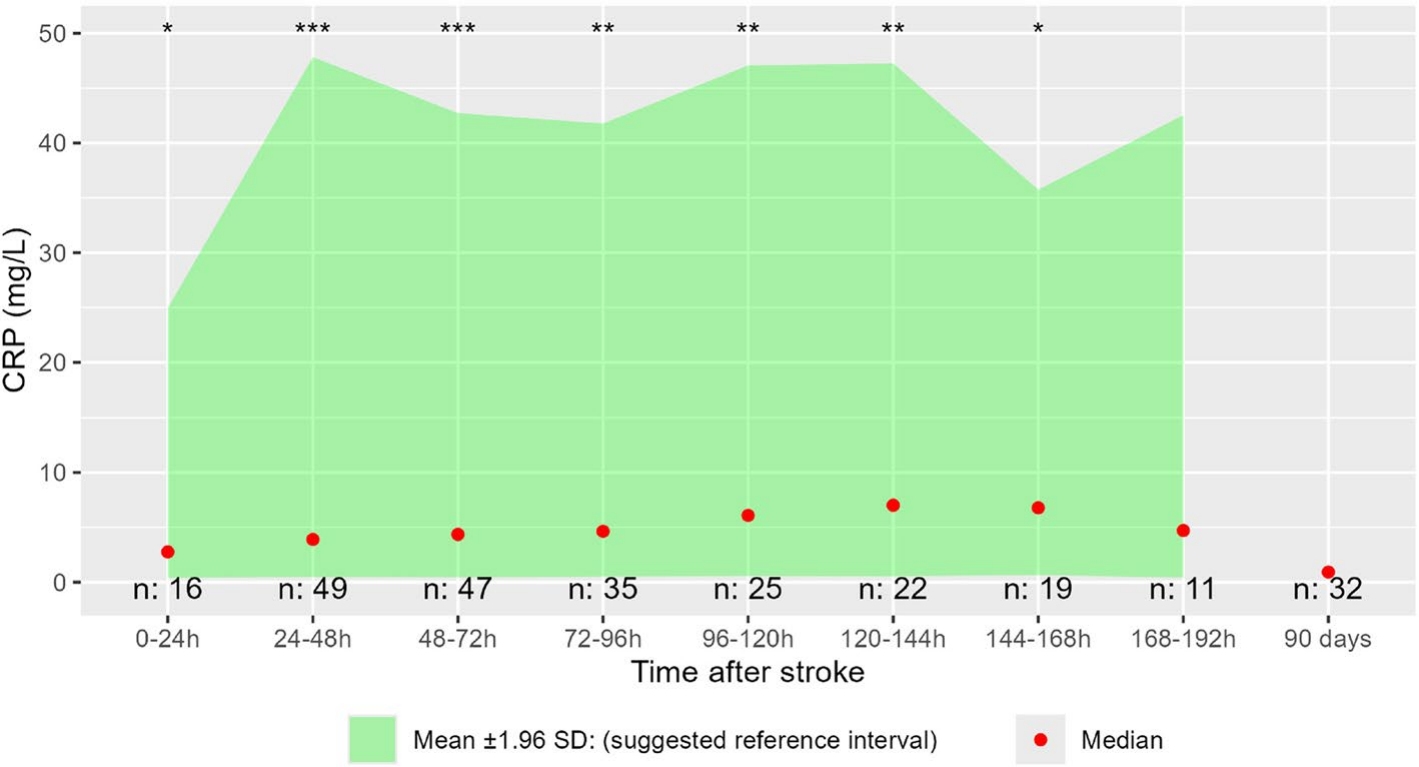
CRP in patients without complications. Difference tested day for day compared to 90-day follow-up using paired T-test on log transformed data, *p*-value: * = < 0,05, ** = < 0,01, *** = < 0,00001. Mean ± 1.96 SD-values calculated from log-transformed data and reverse-transformed for presentation. “n:” is the number of observations from the respective days. *CRP* C-reactive protein

**Fig. 4:**
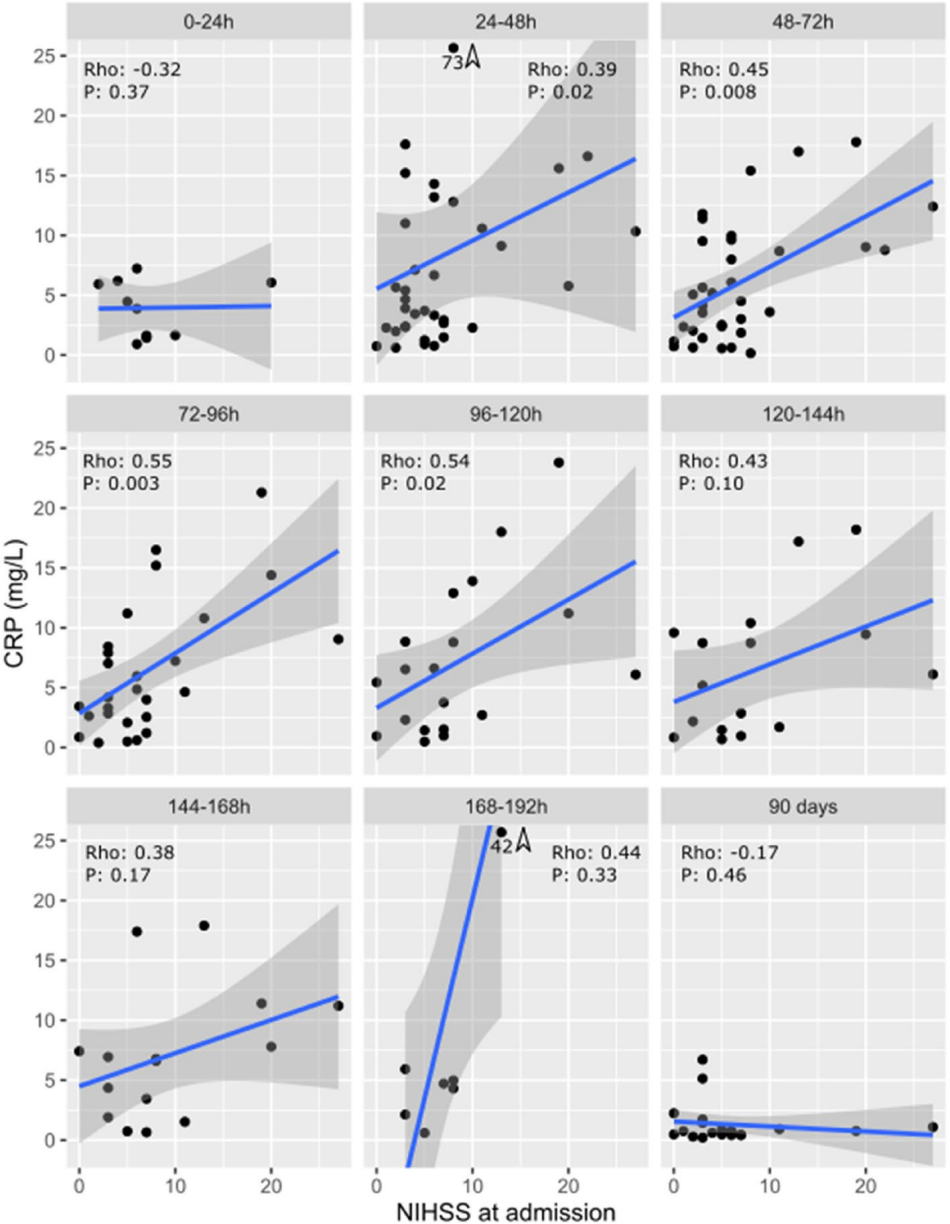
Correlation between NIHSS evaluated at admission and CRP sampled respective days. Lines are linear regressions, and shaded areas are 95% CI of these. Since CRP is not normally distributed, linear regression lines have to be interpreted with caution but are shown for clarity. P- and Rho-values are calculated by Spearman correlation and as such are independent of assumptions of normal distribution. Outliers are indicated at the upper border with arrow and value. NIHSS: National Institutes of Health Stroke Scale, CRP: C-reactive protein

**Fig. 5:**
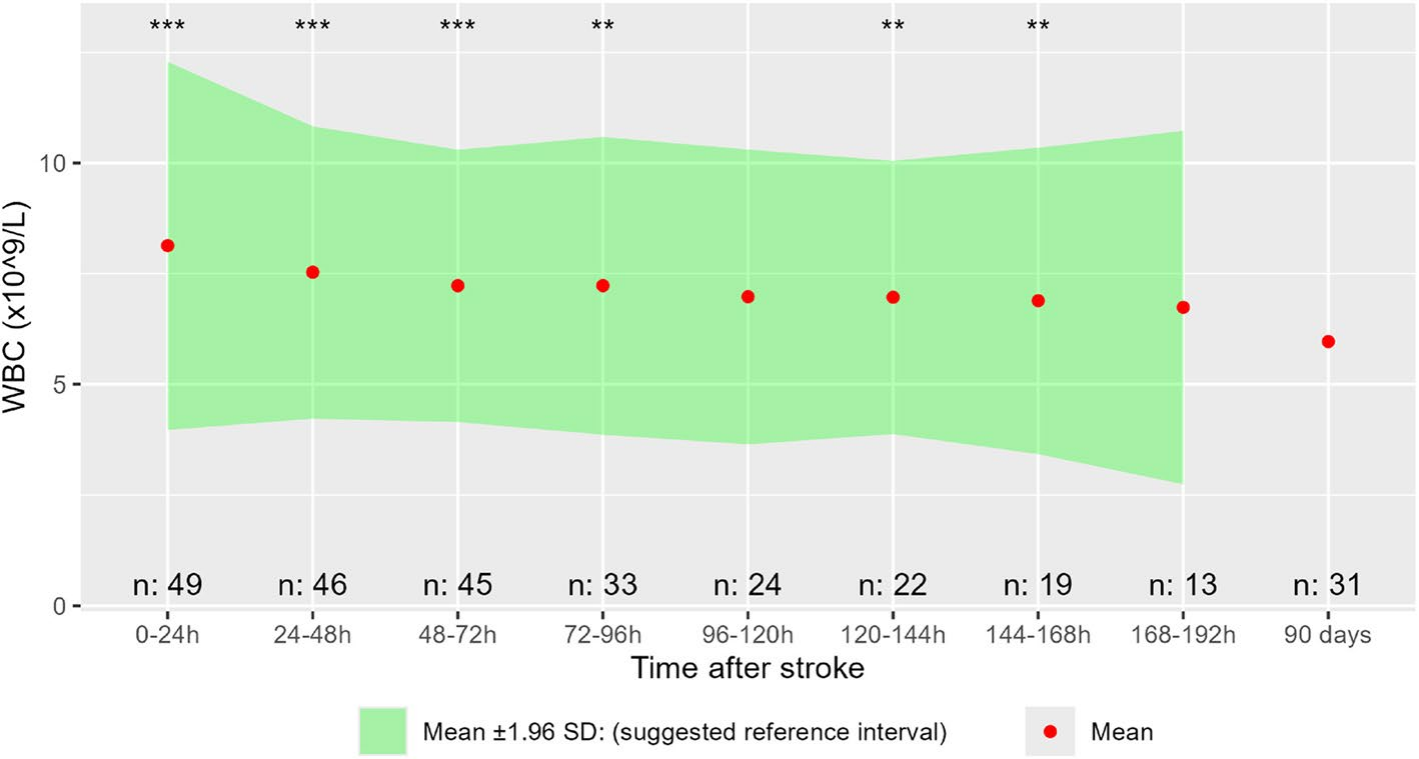
WBC in patients without complications. Difference tested day for day compared to 90-day follow-up using paired T-test, *p*-value: * = < 0,05, ** = < 0,01, *** = < 0,001. “n:” is the number of observations from the respective days. WBC: White Blood Cell count

CRP was slightly elevated during hospitalization compared to 90-day follow up, most significantly at 24–48 and 48-72 h after stroke (Fig. 3). On these days, the upper limits of a computed reference interval (mean + 1.96SD on the log transformed values) were 47.8 mg/L and 42.7 mg/L respectively, meaning that no more than 2.5% of stroke patients without an overt complication can be expected to have a higher CRP value. Median CRP values ranged from 2.8 mg/L in the first 24 h to 7.0 mg/L on 120-144 h after stroke. On follow-up median CRP was 0.9 mg/L. There was a significant correlation between NIHSS at admission to hospital and CRP at 24 to 120 h after stroke onset. (Fig. 4) There was no significant correlation between NIHSS at inclusion in the study and CRP measured at any day. No significant correlation was found between CRP and infarction size or between CRP and size of hemorrhagic stroke (Figs.5–8 in supplemental material).

Apart from NIHSS at admission, only atrial fibrillation was significantly associated with CRP (48–72 h after stroke) in the preceding univariate step for the multivariate analysis. In the multiple regression, no significant association remained for atrial fibrillation while NIHSS at admission remained significant (*p* = 0.041).

### WBC

WBC was elevated during hospitalization compared to 90-day follow up (Fig. 5). This was most pronounced during the first days after stroke. Mean WBC values ranged from 6.74 × 10^9/L on 168-192 h after stroke to 8.13 × 10^9/L in the first 24 h, compared to 5.96 × 10^9/L on follow-up.

There was no correlation between WBC measured at any day and NIHSS, infarction size or size of hemorrhagic stroke (Figs. 9–12 in supplemental material).

For the first, univariate, step of the multiple regression analysis, only sex was even close to being significantly associated with WBC (*p* = 0.094) in the first 24 h after stroke. Because of this, no multiple regression was performed.

## Discussion

In the first days after stroke, there is a significant elevation of body temperature, CRP and WBC in complication-free stroke patients, compared to day 90. A modest elevation in WBC may be seen in a few patients the first 24 h after stroke, and in body temperature and CRP the following 24 h. However, the levels are generally lower than what could be expected from a bacterial infection. [13–15] A complication must be strongly suspected if CRP levels rise above 50 mg/L or temperature reaches 38 °C. For WBC, a slight elevation can be expected the first 24 h where a few patients reach 12 × 10^9/L. After this initial elevation, levels above 11 × 10^9/L are very uncommon.

Median NIHSS at inclusion was 4. While this is less than some other studies [8], it is similar to the median in the Swedish Stroke Registry, 3 [16]. We consider it representative of a general stroke population in a hospital setting under similar circumstances.

Studies aiming to investigate inflammatory parameters in complication-free stroke patients are rare. While Emsley et al. also report on increased levels of CRP and WBC [8], there is a distinct difference in the kinetics, especially concerning WBC. In our study, WBC was increased from the first measurement and decreased after one or two days. In the study by Emsley et al. on the other hand, WBC levels increased during the first days. Interestingly, this increase was less marked after exclusion of patients with infection even if initial values were similarly elevated. This suggests that the initial increase is independent of infections while later elevations could be related to them. The fact that in the current study levels decreased successively, could be a result of a more thorough exclusion of patients with complications. In another study by Marquardt et al. [9], the authors found a similar pattern of WBC with an initial increase and successive normalization. They did not find evidence of an increase in CRP in stroke patients, neither compared to risk factor matched controls nor compared to 90 days post-stroke. Despite this, they did find a correlation between CRP and stroke severity. The current study showed both a highly significant CRP increase compared to 90 days post stroke and a significant correlation between one measure of stroke severity and CRP.

When considering the increases in inflammatory parameters – and supposing that these changes are not caused by infections or venous thrombosis – it is reasonable to believe they are caused either by a direct inflammatory response to the stroke itself or by a hitherto overlooked intermediary condition occurring in at least half of stroke patients.

This overlooked condition, if it exists, must not be something widely known to cause fever, such as an infection, since this study has looked at and excluded all commonly known causes. However, since body temperature seldom exceeds 37.5 °C and CRP seldom exceeds 10 mg/L, it is possible that there are conditions that cause this kind of low-grade inflammation that have not yet been recognized as such. Such a condition or state could, theoretically, be dysphagia (but not pneumonia secondary to dysphagia), being bedbound, or even a treatment, such as intravenous contrast, given to most stroke patients. We experimentally tested to see if there was any correlation between mobility (as documented by research nurse daily) or dysphagia (as tested by ward nurse at admission) and WBC levels but found no correlation (Figs. 13–14 in supplemental material). We chose WBC for this experimental analysis since we found CRP and body temperature was correlated to expected risk factors such as stroke severity or type. WBC on the other hand seemed to be completely uncorrelated to known risk factors, why we expanded the search.

Another hypothesis is that a stroke can directly perturb the central nervous system’s orchestration of the inflammatory system by affecting some unknown “inflammatory center”. While the location or existence of such a location is unknown, the fact that no single area was affected in more than 31% of patients and since the data suggests more than half of patients had increased body temperatures, this seems unlikely as a sole cause.

Multiple regression analysis did not reveal any significant correlation between inflammatory parameters and comorbidities, stroke size, infarct location, age or sex. A tendency towards slightly more elevated body temperatures, but not CRP or WBC, was seen among patients with cardioembolic strokes. This association was unexpected, and the results from the multiple regression should be regarded as exploratory and interpreted with great caution, even more since the number of patients restricted the power of the analysis. One could speculate that patients with cardioembolic strokes might have emboli in other parts of the body, and that the inflammatory response caused by the embolic load could increase the body temperature.

If it was a direct effect of the tissue damage of the stroke itself, one would expect to see a strong correlation between stroke size and level of inflammation. We only found a statistically significant correlation between stroke size measured as NIHSS and CRP in some of the days. Even though body temperature showed some (statistically insignificant) correlation to stroke size, there is no correlation between stroke size and WBC at all. As in all studies seeking to investigate a complication free population, it is hard to rule out that in some patients a mild, subclinical infection has been overlooked and that these patients are unintentionally included in the analysis. However, our study design has several advantages over previous ones due to rigorous protocol and prospective inclusion. Additionally, for most analyses, a few patients with subclinical infections erroneously included in the analyses will not have had a major effect on the results. It is highly unlikely that the significant difference compared to follow-up values is a result of this. Considering temperature, on the first measured day more than half of all patients had temperatures above 37.0 °C, while at day 90, only one did. Only if almost half the patients had a subclinical infection could you expect this result to be an effect of such infections. Considering CRP, the difference is similar. On day 2, 80% have a CRP over 2 and on day 90 only 22% do.

Another issue is multiple testing increasing the risk of a type I error. Since a lot of the tests are strongly positively correlated to each other, either time-series data or testing similar concepts using different measurements (e.g. stroke severity measured as NIHSS or infarction size measured on MRI), a Bonferroni correction would be overly conservative.

## Conclusions

Stroke is associated with an increase in body temperature and common blood markers of inflammation in patients without overt complications. However, the increases are modest and the correlation between these elevations and the size of the stroke is small. Our results suggest that CRP levels above 50 mg/L, body temperature above 38 °C or WBC above 11 × 10^9/L (or above 12 × 10^9/L the first 24 h after stroke) are highly unlikely to be an effect of stroke itself and should be suspected to be signs of a complication, most commonly a bacterial infection.

## Supplementary Information

Supplementary Material 1

## Data Availability

The datasets generated during the current study are available from the corresponding author on reasonable request.

## Abbreviations

CRP: C-reactive protein
CT: Computed tomography
DVT: Deep vein thrombosis
DWI: Diffusion weighted images
hsCRP: High sensitivity CRP
MRI: Magnetic resonance imaging
NIHSS: National Institutes of Health Stroke Scale
TE: Echo time
TIA: Transient ischemic attack
TOAST: Trial of Org 10,172 in Acute Stroke Treatment
TR: Repetition time
UTI: Urinary tract infections
WBC: White blood cell count/Leucocyte particle concentration
