# Supplementary material for: Inflammatory response after stroke—A clinical observation study
Source: BMC Neurol. 2025 May 30;25:233. doi: 10.1186/s12883-025-04244-y (PMC12125930; doi:10.1186/s12883-025-04244-y)
Supplement: Supplementary file 1 — Supplementary Material 1 [file 12883_2025_4244_MOESM1_ESM.docx]

Supplemental material

Table of contents:

[Body temperature 2](#_Toc197802380)

[CRP 6](#_Toc197802381)

[WBC 10](#_Toc197802382)

[Experimental correlations with WBC 14](#_Toc197802383)

[Daily questions and examinations 15](#_Toc197802384)

[Clinical examinations 15](#_Toc197802385)

[Questionnaire 15](#_Toc197802386)

[Criteria for complications 17](#_Toc197802387)

[Clarification of exclusion criteria 18](#_Toc197802388)

[Additional information on patients excluded from analysis on the basis of complications 19](#_Toc197802389)

[CRP 19](#_Toc197802390)

[WBC 21](#_Toc197802391)

[Body temperature 21](#_Toc197802392)

# Body temperature


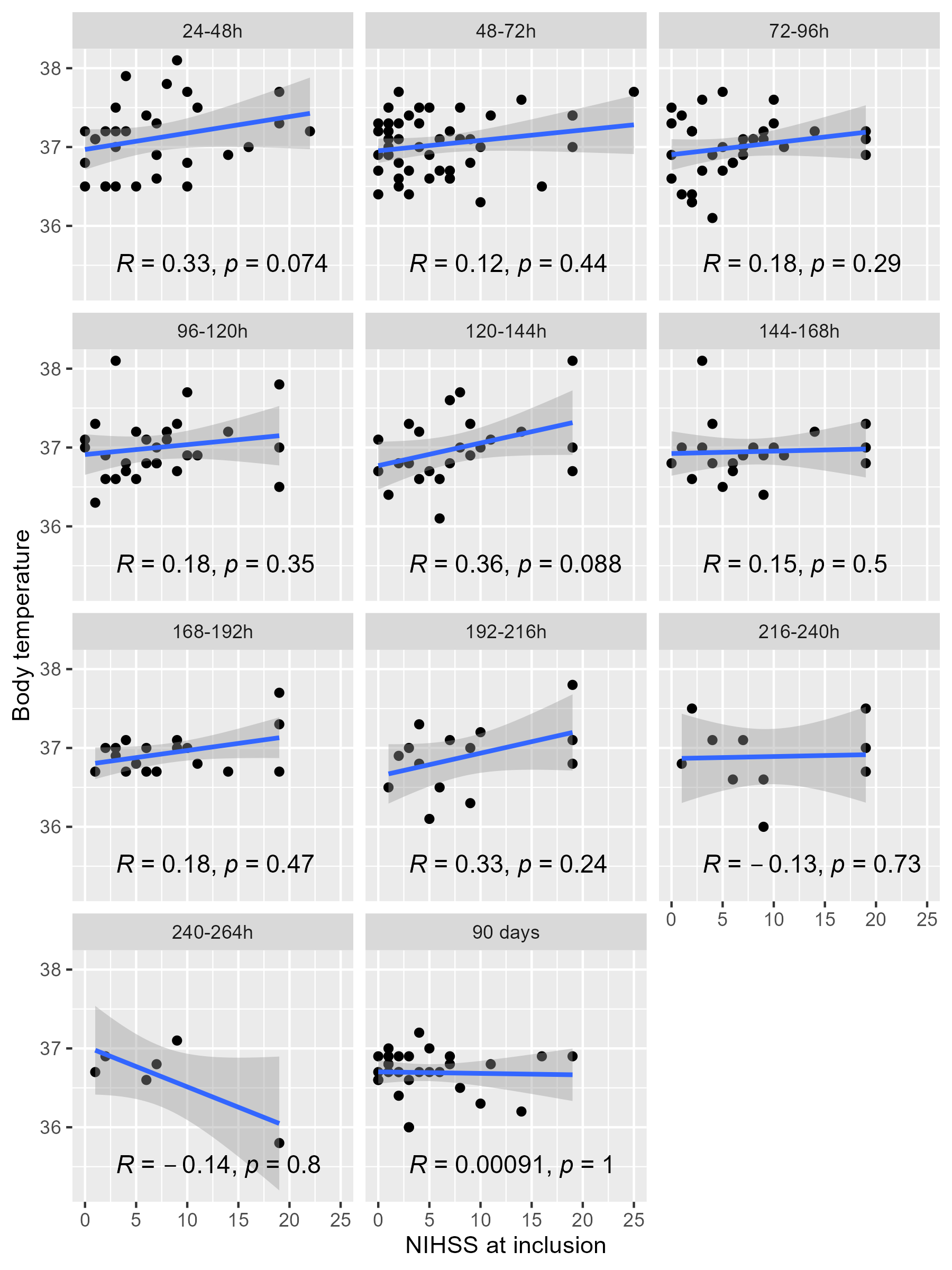


**Figure 1 - Body temperature correlated to NIHSS at inclusion.** Blue lines are linear regressions, and shaded areas are 95% CI of these. Since NIHSS is not normally distributed, linear regression lines have to be interpreted with caution but are shown for clarity. P- and R-values are calculated by Spearman correlation and as such are independent of assumptions of normal distribution. NIHSS: National Institutes of Health Stroke Scale


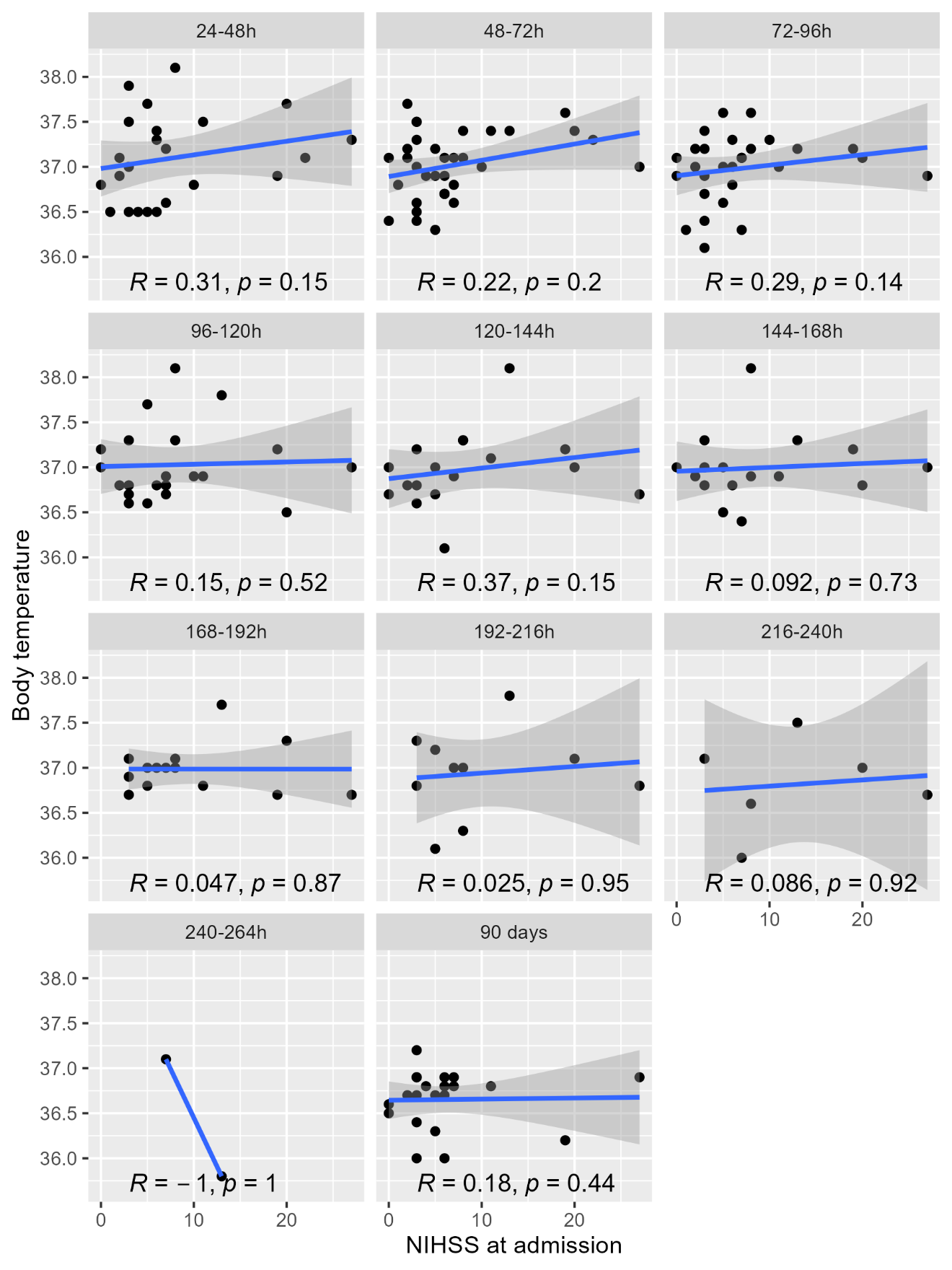


**Figure 2 - Body temperature correlated to NIHSS at admission.** Blue lines are linear regressions, and shaded areas are 95% CI of these. Since NIHSS is not normally distributed, linear regression lines have to be interpreted with caution but are shown for clarity. P- and R-values are calculated by Spearman correlation and as such are independent of assumptions of normal distribution. NIHSS: National Institutes of Health Stroke Scale


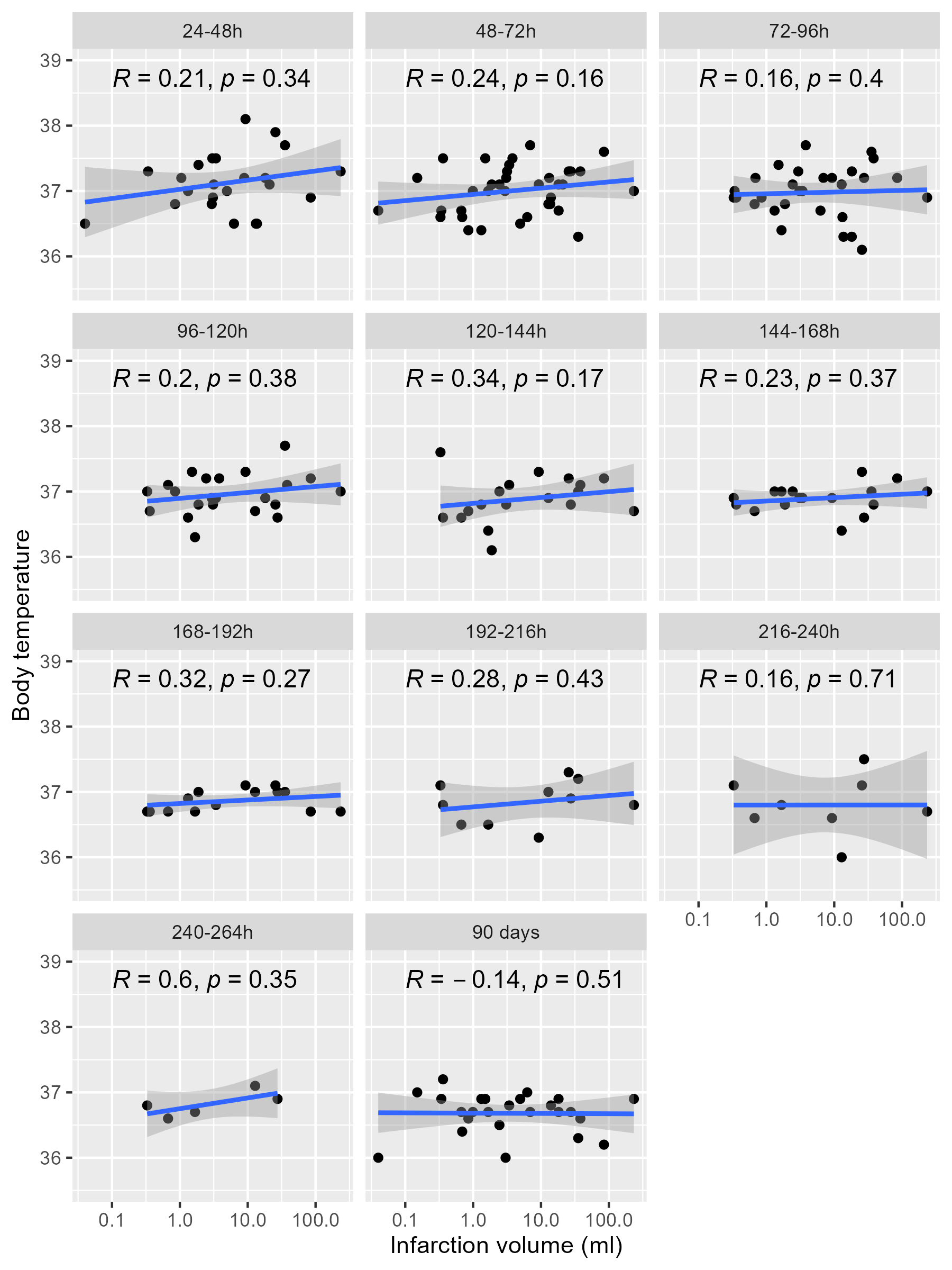


**Figure 3 -** **Body temperature correlated to infarct volume (log).** Blue lines are linear regressions, and shaded areas are 95% CI of these. Since infarct volume is not normally distributed, linear regression lines have to be interpreted with caution but are shown for clarity. P- and R-values are calculated by Spearman correlation and as such are independent of assumptions of normal distribution.


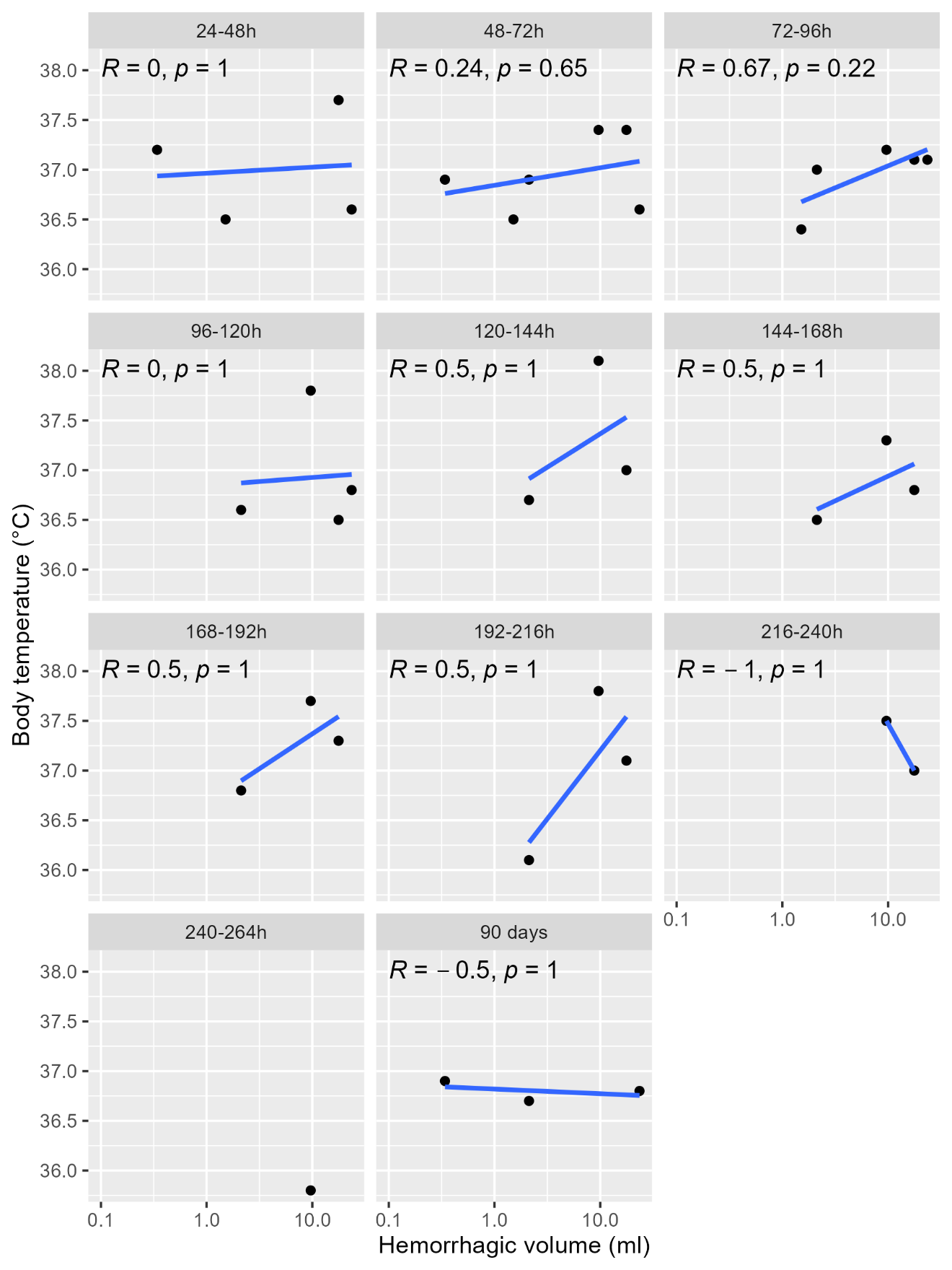


**Figure 4 -** **Body temperature correlated to hemorrhagic volume (log).** Blue lines are linear regressions. Since hemorrhagic volume is not normally distributed, linear regression lines have to be interpreted with caution but are shown for clarity. P- and R-values are calculated by Spearman correlation and as such are independent of assumptions of normal distribution.

# CRP


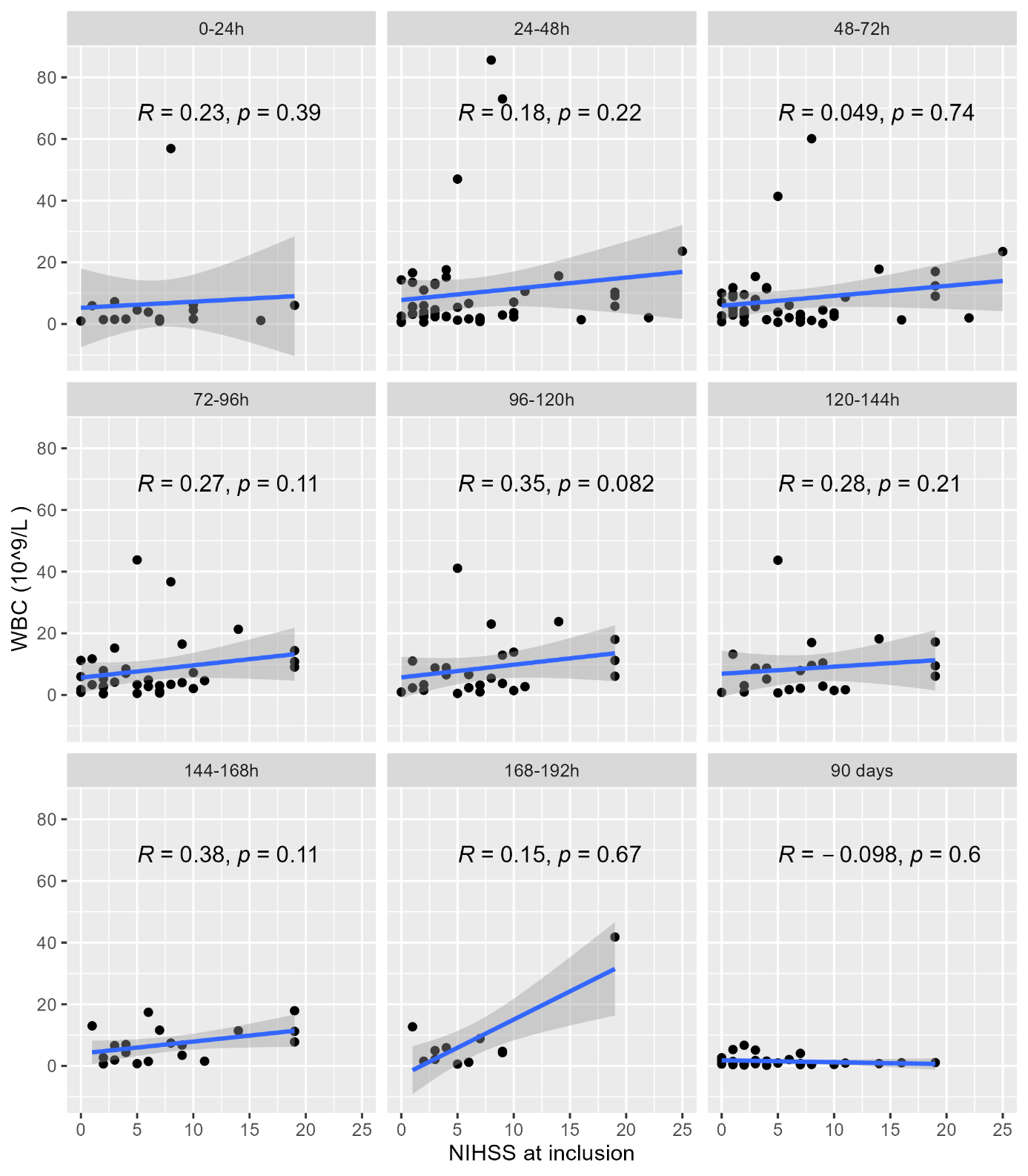


**Figure 5** - **CRP correlated to NIHSS at inclusion.** Blue lines are linear regressions, and shaded areas are 95% CI of these. Since NIHSS and CRP are not normally distributed, linear regression lines have to be interpreted with caution but are shown for clarity. P- and R-values are calculated by Spearman correlation and as such are independent of assumptions of normal distribution. NIHSS: National Institutes of Health Stroke Scale, CRP: C-reactive protein


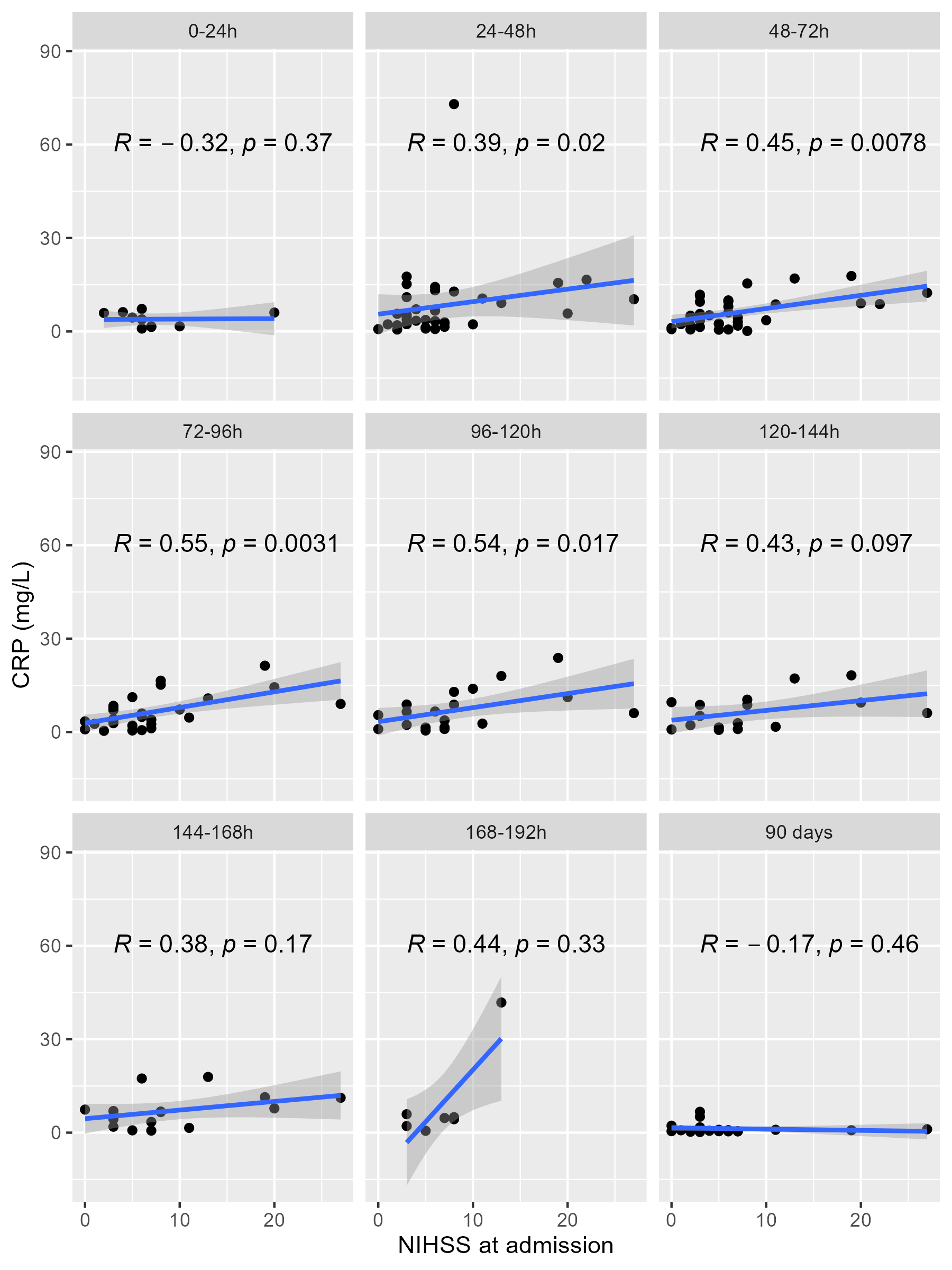


**Figure 6 -** **CRP correlated to NIHSS at admission.** Similar to graph in main article but zoomed out to also include outliers. Blue lines are linear regressions, and shaded areas are 95% CI of these. Since NIHSS and CRP are not normally distributed, linear regression lines have to be interpreted with caution but are shown for clarity. P- and R-values are calculated by Spearman correlation and as such are independent of assumptions of normal distribution. NIHSS: National Institutes of Health Stroke Scale, CRP: C-reactive protein


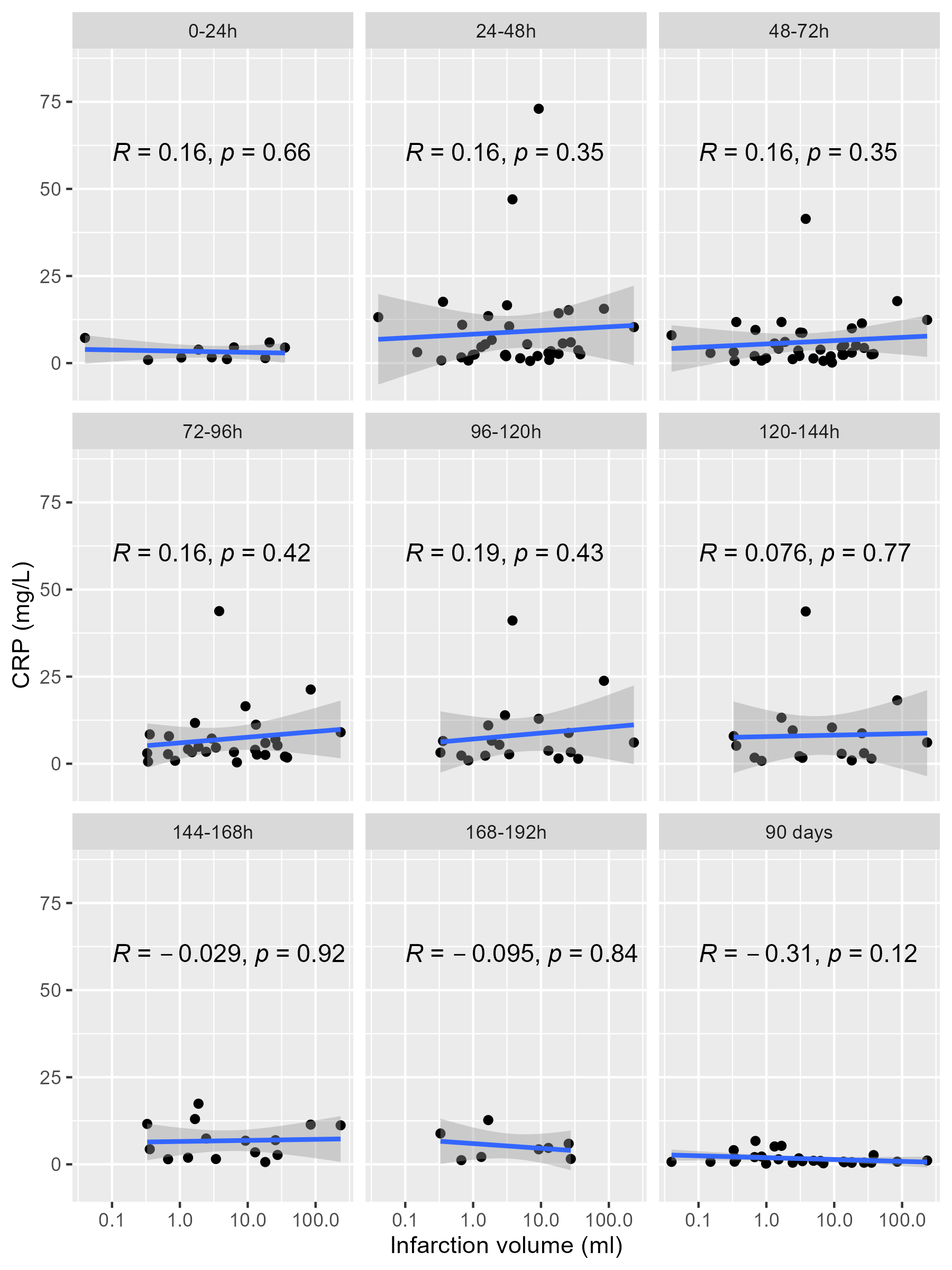


**Figure 7 -** **CRP correlated to infarct volume (log)**. Blue lines are linear regressions, and shaded areas are 95% CI of these. Since CRP is not normally distributed, linear regression lines have to be interpreted with caution but are shown for clarity. P- and R-values are calculated by Spearman correlation and as such are independent of assumptions of normal distribution. CRP: C-reactive protein


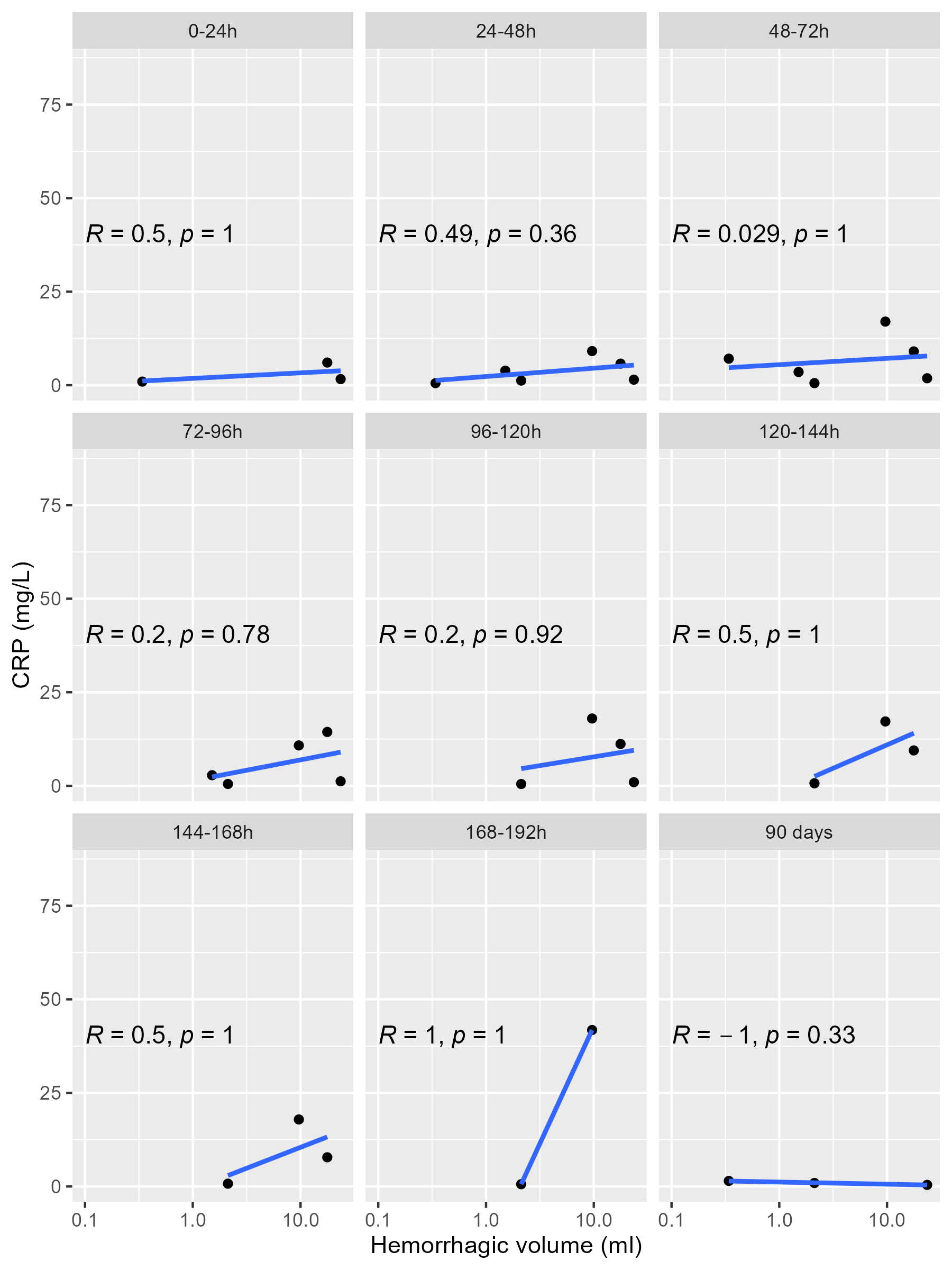


Figure 8 - **CRP correlated to hemorrhagic volume (log)**. Blue lines are linear regressions. Since CRP is not normally distributed, linear regression lines have to be interpreted with caution but are shown for clarity. P- and R-values are calculated by Spearman correlation and as such are independent of assumptions of normal distribution. CRP: C-reactive protein

# WBC


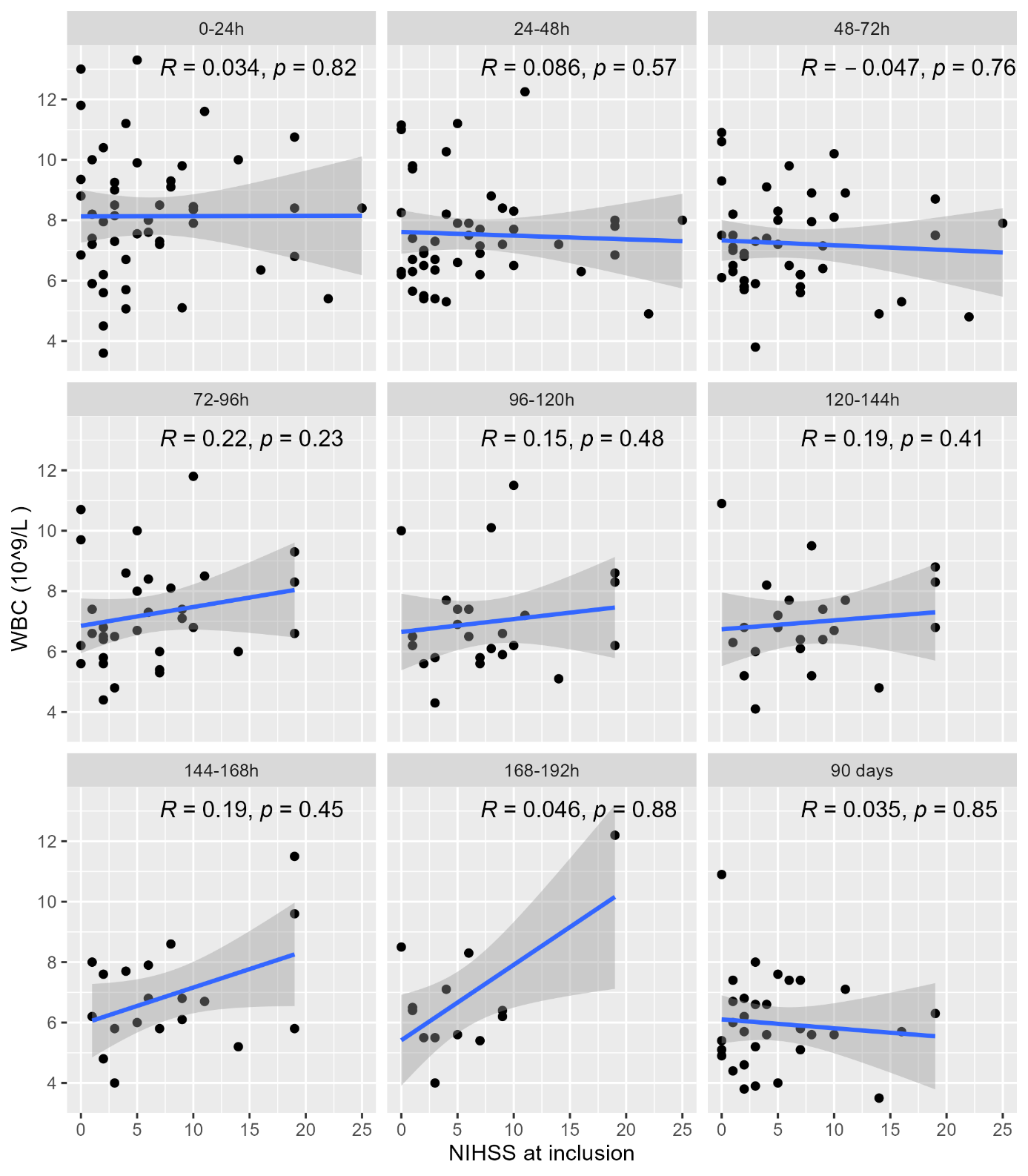


**Figure 9 – WBC correlated to NIHSS at inclusion.** Blue lines are linear regressions, and shaded areas are 95% CI of these. Since NIHSS is not normally distributed, linear regression lines have to be interpreted with caution but are shown for clarity. P- and R-values are calculated by Spearman correlation and as such are independent of assumptions of normal distribution. NIHSS: National Institutes of Health Stroke Scale, WBC: White blood cell count


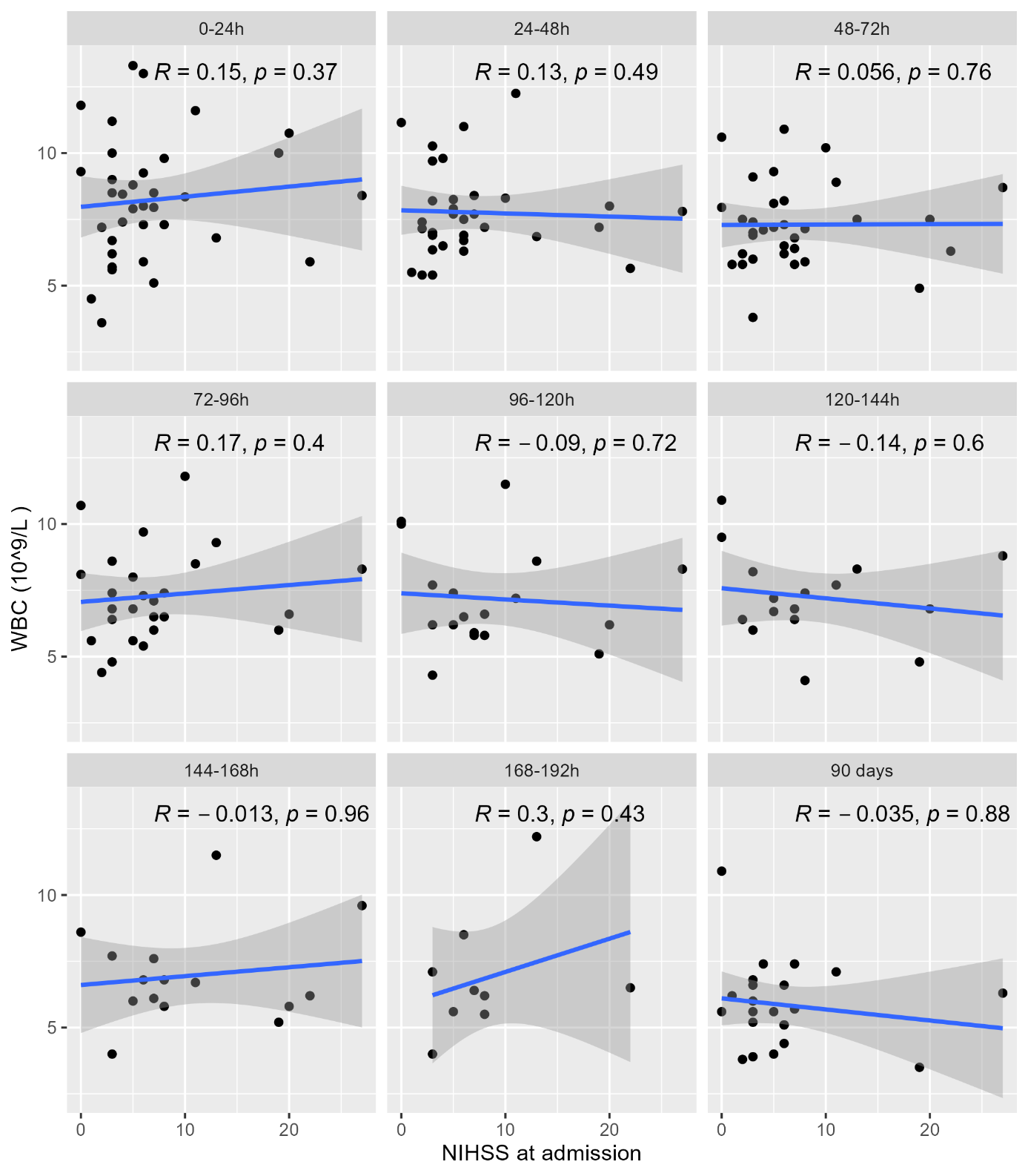


**Figure 10 - WBC correlated to NIHSS at admission.** Blue lines are linear regressions, and shaded areas are 95% CI of these. Since NIHSS is not normally distributed, linear regression lines have to be interpreted with caution but are shown for clarity. P- and R-values are calculated by Spearman correlation and as such are independent of assumptions of normal distribution. NIHSS: National Institutes of Health Stroke Scale, WBC: White blood cell count


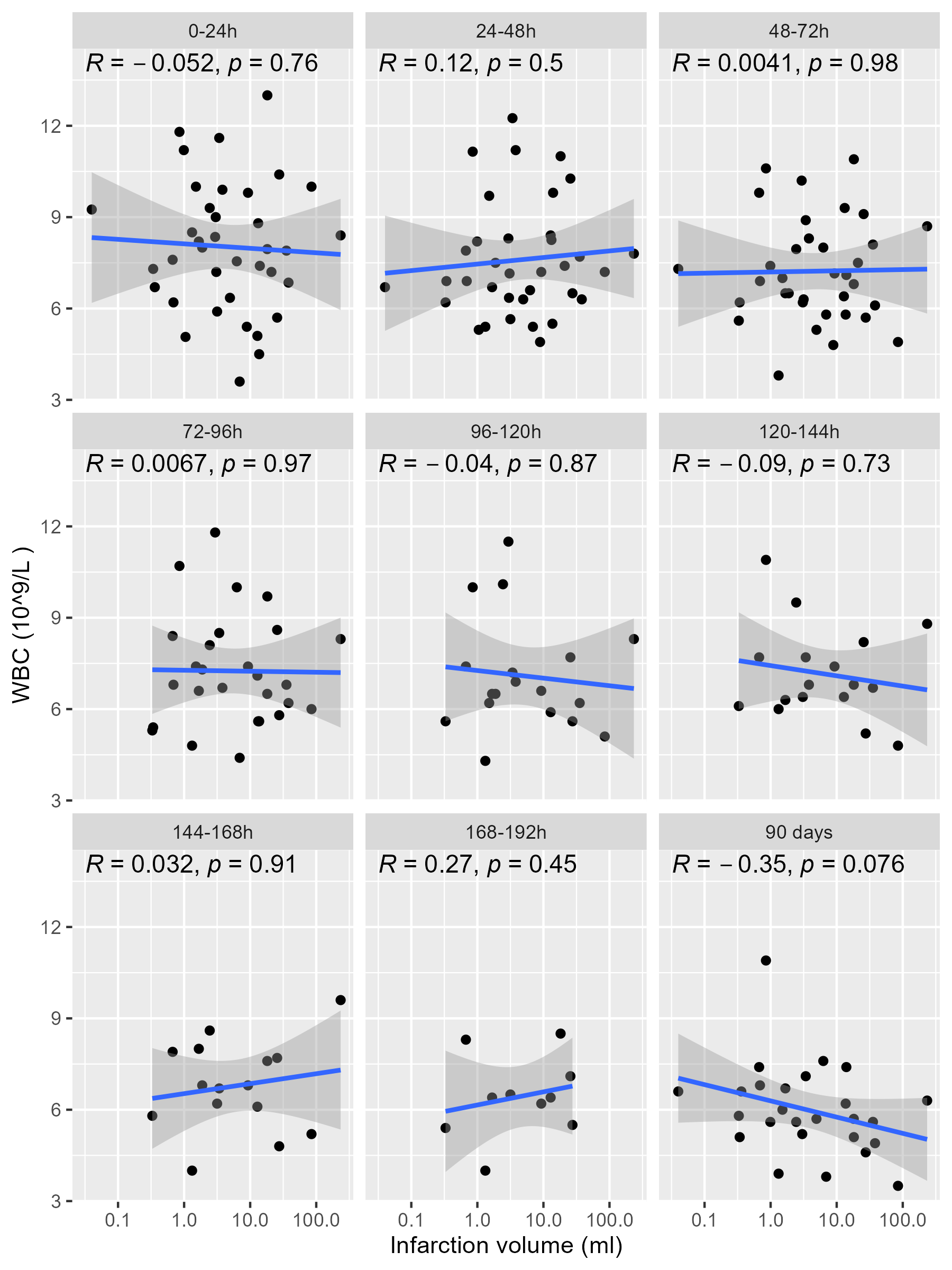


**Figure 11 - WBC correlated to infarct volume (log).** Blue lines are linear regressions, and shaded areas are 95% CI of these. Since infarct volume is not normally distributed, linear regression lines have to be interpreted with caution but are shown for clarity. P- and R-values are calculated by Spearman correlation and as such are independent of assumptions of normal distribution. WBC: White blood cell count


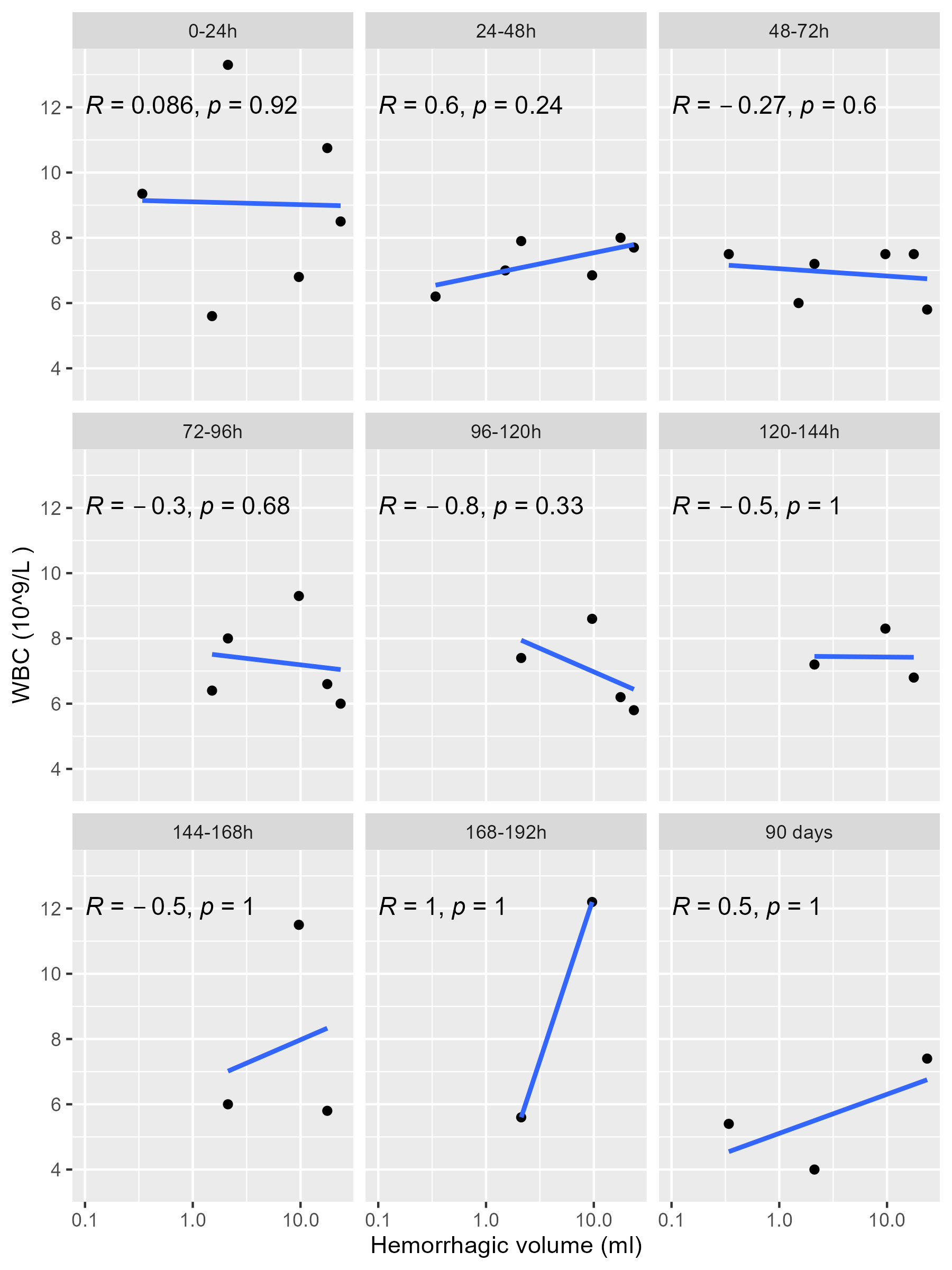


**Figure 12 - WBC correlated to hemorrhagic volume (log).** Blue lines are linear regressions. Since hemorrhagic volume is not normally distributed, linear regression lines have to be interpreted with caution but are shown for clarity. P- and R-values are calculated by Spearman correlation and as such are independent of assumptions of normal distribution. WBC: White blood cell count

# Experimental correlations with WBC


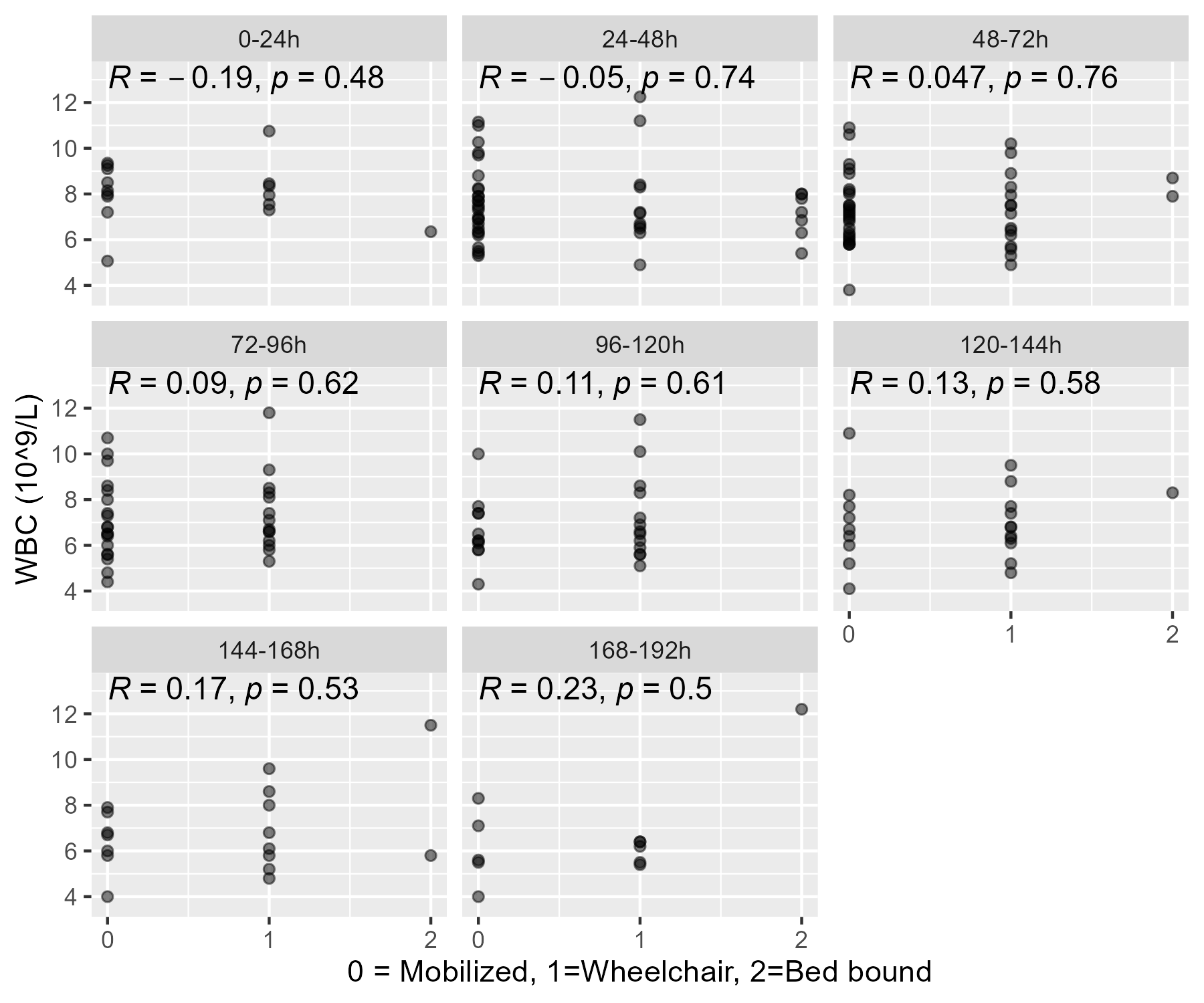


**Figure 13 – WBC correlated to mobility.** WBC: White blood cell count, R: Spearman’s rho, p: p-value of spearman correlation


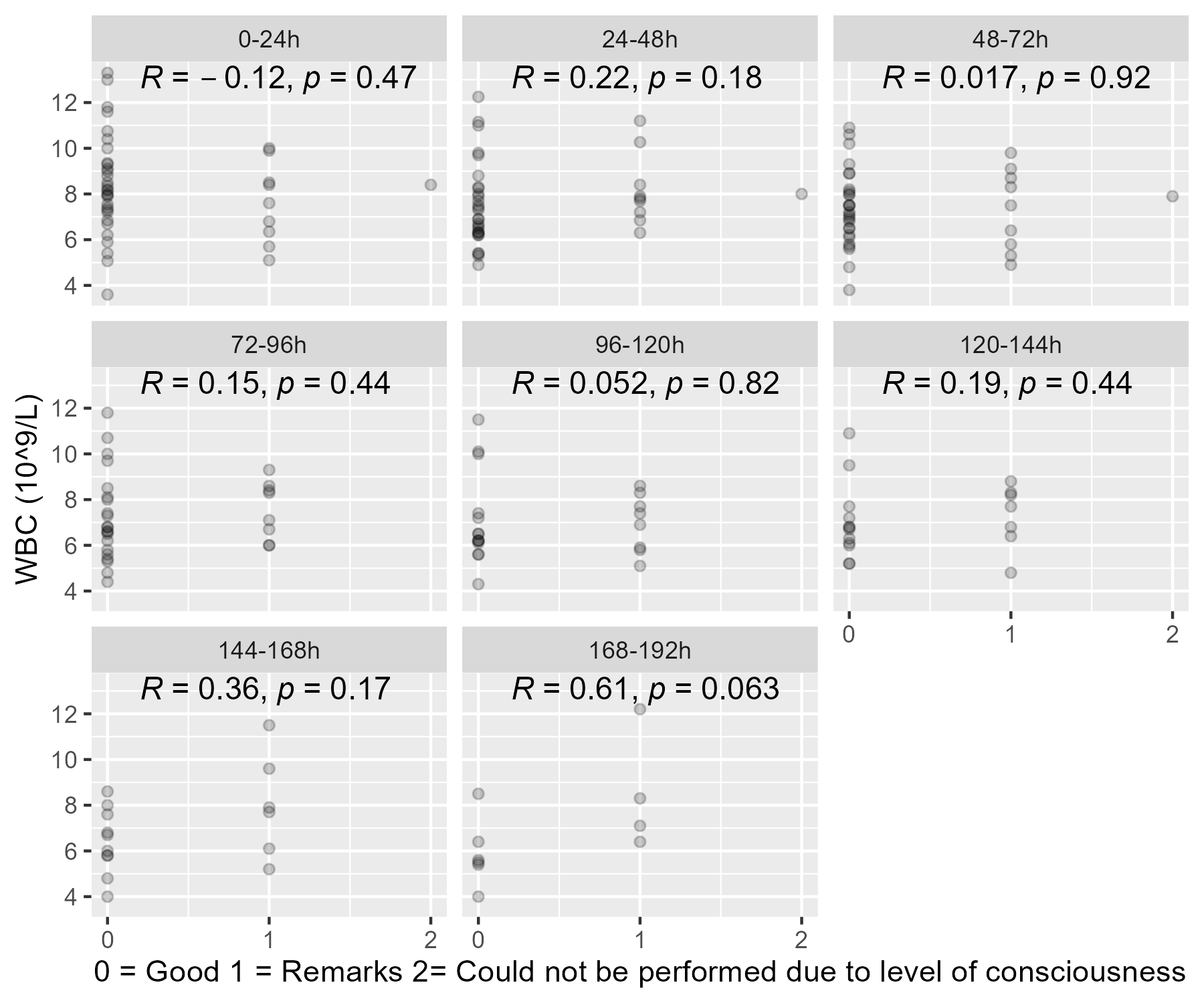


**Figure 14** - **WBC correlated to swallow test for dysphagia.** WBC: White blood cell count, R: Spearman’s rho, p: p-value of spearman correlation

# Daily questions and examinations

### Clinical examinations

- Morning temperature in degrees Celsius, measured with a rectal thermometer.
- Oxygen saturation, measured by pulse oximetry.
- Respiratory rate
- Blood pressure
- Lower leg circumference and palpation for soreness or pain in the lower leg
- Inspection of pressure ulcers, wounds, and intravenous lines

### Questionnaire

*Answered by the patient, if conscious and able to make themself understood, otherwise assessed and answered by the ward nurse.*

1. Do you have cough? *(Yes/ No/ Don’t know)*
   1. If *YES*, do you have heavy cough? *(Yes/ No/ Don’t know)*
   2. If *YES*, is it of new onset, worsened character, or unchanged character? *(New/ Worsened/ Unchanged/ Don’t know)*
2. Do you cough up sputum or fluid? *(Yes/ No/ Don’t know)*
   1. If *YES,* do you cough up a lot of sputum or fluid? *(Yes/ No/ Don’t know)*
   2. If *YES*, has the sputum or fluid worsened in amount, or changed in character? *(Yes/ No)*
3. Do you feel out of breath?
   1. If *YES,* do you have severe breathlessness? *(Yes/ No)*
   2. If *YES*, is it of new onset, worsened character, or unchanged character? *(New/ Worsened/ Unchanged)*
4. Do you have chest pain? *(Yes/ No)*
   1. If YES, do you have severe breast pain? *(Yes/ No)*
5. Do you have abdominal pain? *(Yes/ No)*
   1. If YES, do you have severe abdominal pain? *(Yes/ No)*
6. Does it sting or burn when you urinate? *(Yes/ No/ Uses a catheter)*
7. Do you need to urinate more frequently than usual? *(Yes/ No)*
8. Do you have a feeling of illness or pending infection (malaise)? *(Yes/ No)*

*Answered by the ward nurse.*

1. Was the questionnaire answered today? *(Yes/ No)*
2. Did the patient vomit during the last 24 hours? *(Yes/ No)*
3. Did the patient experience any diarrhea during the last 24 hours? *(Yes/ No)*
4. Is the patient upright and able to move around, mobilized using a wheelchair or confined to bed? *(Upright/ Wheelchair/ Bed-bound)*
5. If the patient is intubated, have you had to suction the tube more frequently during the last 24 hours? *(Yes/ No/ Don’t know)*
6. Does the patient seem confused? *(Yes/ No)*
   1. If *YES*, is the confusion of new onset, worsened character, or unchanged character? *(New/ Worsened/ Unchanged)*
7. Does the patient require oxygen? *(Yes/ No)*
   1. If *YES*, how many liters?

# Criteria for complications

Criteria for exclusion due to complications

*A) Meeting ≥ 1 of the following criteria during hospital stay (up to 10 days from onset of stroke symptoms)*

- Diagnosed with one of the following complications by the treating physician.
  1. Bacterial Respiratory Tract Infection
  2. Upper Urinary Tract Infection (UTI)
  3. Sepsis
  4. Deep Venous Thrombosis (DVT)
  5. Gastroenteritis
  6. Infected pressure ulcer, wound or intravenous line.
- Received antibiotic treatment.

*B) Meeting ≥ 1 of the following criteria during hospital stay (up to 10 days from onset of stroke symptoms)*

- Signs of Respiratory Tract Infection, with ≥ 2 of:
  1. New onset or worsening cough with duration ≥2 days; or new onset or worsening dyspnea with duration ≥2 days; or tachypnea >25 breaths per minute
  2. Saturation < 85% or need for oxygen.
  3. New onset or change in character of sputum.
  4. Unilaterally decreased breath sounds at lung auscultation.
- Signs of Upper UTI with C reactive protein (CRP) >30 and ≥ 1 of:
  1. ≥ 2 days of dysuria and/or increased urination frequency
  2. Significant bacteriuria findings, meaning 10^5^ CFU/mL without catheter and 10^6^ CFU/mL with catheter.
  3. Leukocyturia ≥ 2+
- Signs of DVT
  1. Unilateral pain and swelling of the lower leg, both symptoms occurring the same day(s). Swollen leg measuring ≥ 3 cm larger in circumference compared to the other leg. Confirmation by duplex ultrasound.
  2. Bilateral pain and swelling of legs, both symptoms occurring the same day(s). Both legs measuring ≥3 cm larger in circumference compared to other days. Confirmation by duplex ultrasound.
- Signs of gastroenteritis
  - Symptoms of diarrhea, using the WHO definition of more than or equal to four bowel movements per day, AND
  - Vomiting, at least once
  - Both symptoms occurring the same day, one day sufficient for diagnosis.
  - Bacterial culture or viral PCR is NOT needed for diagnosis.
- Signs of infected pressure ulcers, wounds, or intravenous lines.
  - Based on daily inspections and assessments, registered in the study protocol.
  - Regarding pressure ulcers, Mild redness alone, reflecting a European Pressure Ulcer Advisory Panel (EPUAP) pressure ulcer grade 1, does not qualify while all higher grades, regardless of infection status, are included.
  - Traumatic or surgical wounds are not included as long as they show signs of a normal healing process and no clear signs of infection.
  - Intravenous lines with a minor redness or irritation are not included. Likewise are small indurations, indicative of a minor thrombophlebitis not included. Lines, especially central lines, with a larger red area or which are purulent are included in the diagnosis.
  - For practical reasons, the definition of infected or not was hard to distinctively define and an individual assessment was made by the main author from case to case.

# Clarification of exclusion criteria

1. Blood sampling not possible within 48 hours of start of symptoms
   1. Motivated by the importance of the study being able to describe the early inflammatory response.
2. Transitory ischemic attack
   1. This was based on clinical criteria with complete resolution of symptoms within 24 hours.
3. Hemoglobin level below 90 g/l
   1. To assure safety of participants considering the comprehensive and repeated blood sampling.
4. Preexisting active inflammatory illness
   1. Pre-defined instructions included:
      1. Rheumatic diseases such as rheumatoid arthritis (but not osteoarthritis) or polymyalgia rheumatica with active disease in the last two months
      2. use of systemic but not topical or inhaled corticosteroids
      3. Inflammatory bowel disease such as ulcerative colitis or Crohn’s disease with active disease in the last two months.
      4. Abdominal inflammation such as cholecystitis or pancreatitis in the last two months.
   2. Inconclusive cases were arbitrated by the study doctors, authors JS and BR.

# Additional information on patients excluded from analysis on the basis of complications

As described in the manuscript, patients participated in the study regardless of if they had been diagnosed with a complication or not. Since the goal was to describe the inflammatory response caused by stroke and not by complications, patients with a complication were retrospectively removed from analysis. For a more complete understanding of the cohort, data on these patients is presented here. Baseline characteristics are presented in table 1.

|  | Total (n= 19) | Ischemic (n=12) | Hemorrhagic (n= 7) |
| --- | --- | --- | --- |
| Mean age | 77.7 | 79.2 | 75.3 |
| Women | 12 (63%) | 8(67%) | 4(57%) |
| Median NIHSS at admission | 5.5 (n=14) | 4 (n=9) | 7 (n=5) |
| Median NIHSS at inclusion | 11 | 8 | 16 |
| Underwent MRI examination |  | 92% |  |
| Median infarction volume |  | 6.7 ml |  |
| Median hemorrhage volume |  |  | 5.8 ml |
| Hypertension | 89% | 83% | 100% |
| Hypercholesterolemia | 32% | 42% | 14% |
| Atrial fibrillation | 58% | 58% | 57% |
| Diabetes | 16% | 8% | 29% |
| Ischemic heart disease | 5% | 0% | 14% |
| Obstructive lung disease | 16% | 8% | 29% |
| Current smoker | 0% | 0% | 0% |
| Former smoker | 26% | 17% | 43% |
| Attended follow-up visit | 37% | 25% | 57% |
| 1-year mortality | 32% | 42% | 14% |
| Length of stay (median days) | 15 | 14 | 16 |
| Beta-blocker treatment | 79% | 67% | 100% |
| Acetaminophen treatment (regularly) | 26% | 25% | 29% |
| Thrombectomy | 5% | 8% |  |
| Thrombolysis | 5% | 8% |  |

**Table 1:** Population characteristics. NIHSS: National Institutes of Health Stroke Scale

## CRP

As expected, CRP and the suggested reference interval was elevated compared to patients without complications (No statistical comparison made, Figure 15). The suggested interval +-1.96SD is so high that the changes in median CRP are hard to discriminate. Therefore, a zoomed in version is presented as Figure 16.


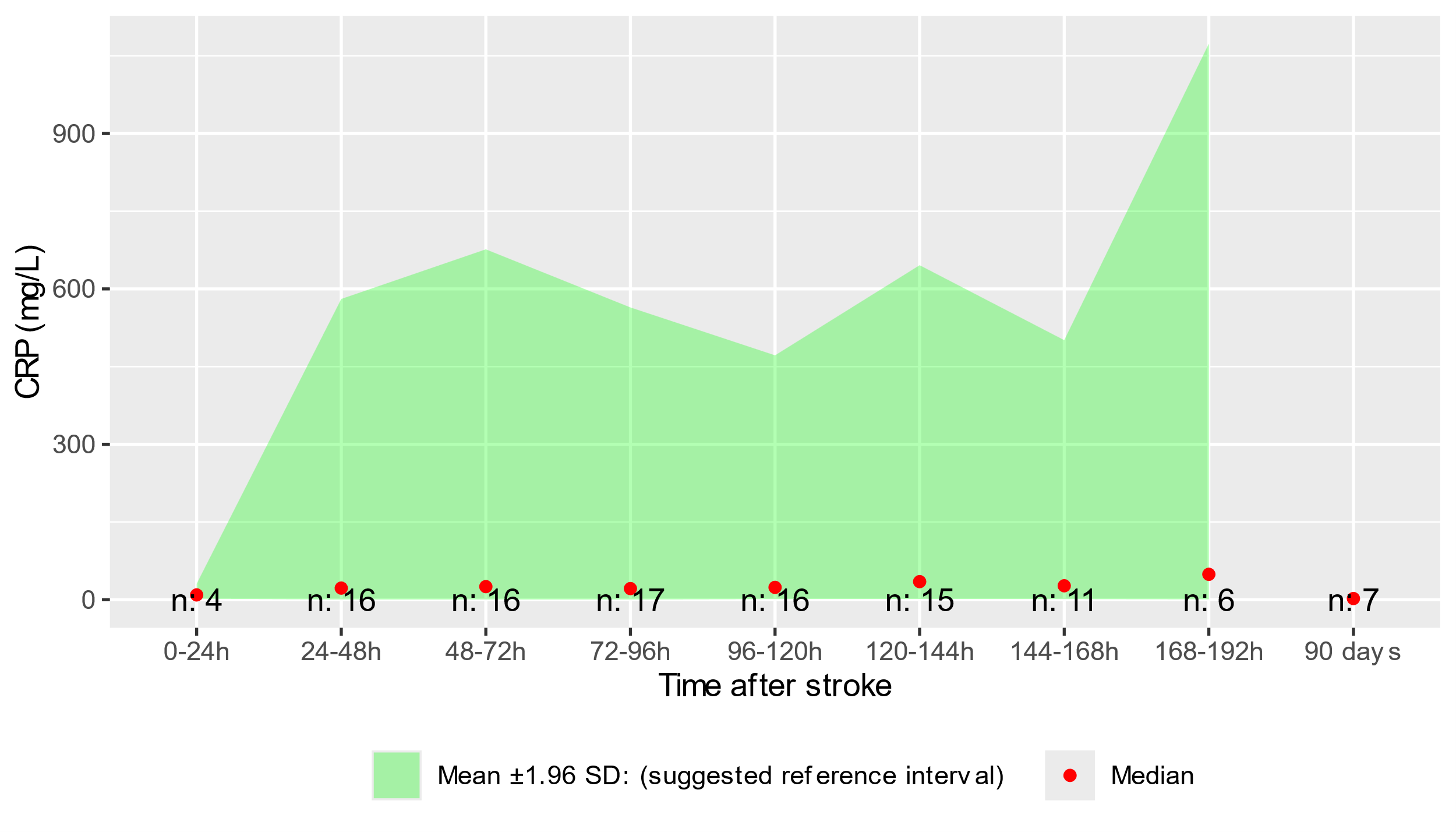


**Figure 15: CRP in patients with** **complications.** Mean ±1.96 SD-values calculated from log-transformed data and reverse-transformed for presentation. “n:” is the number of observations from the respective days. CRP: C-reactive protein.


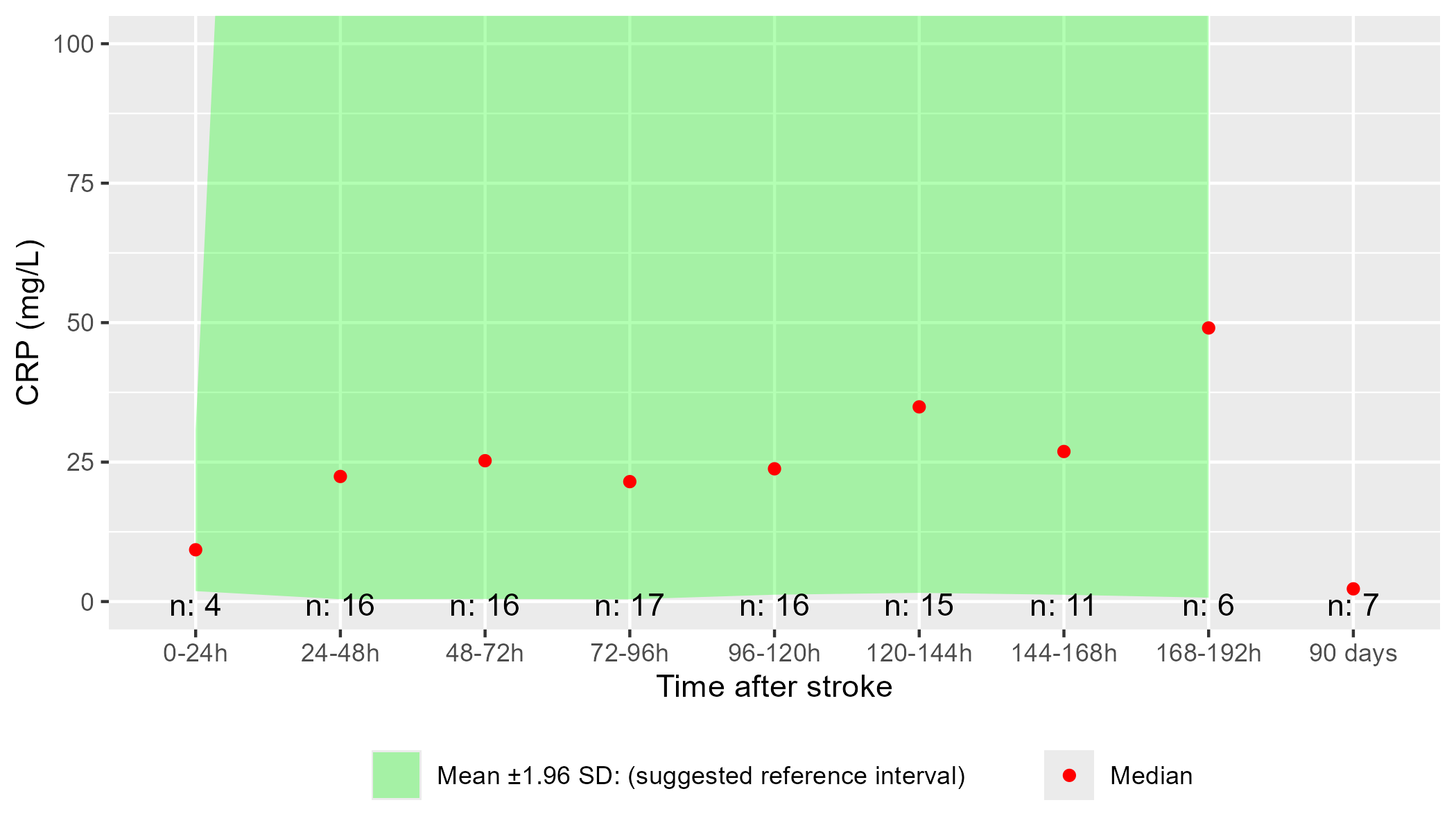


**Figure 16: CRP in patients with complications**. This is figure 15 but with the y-axis zoomed in to highlight changes in median levels. Mean ±1.96 SD-values calculated from log-transformed data and reverse-transformed for presentation. “n:” is the number of observations from the respective days. CRP: C-reactive protein.

## WBC

For WBC a similar but less pronounced pattern is visible. For patients without complications, the increase was highest in the first 24 hours and then decreased day for day. For patients with complications this was not the case.
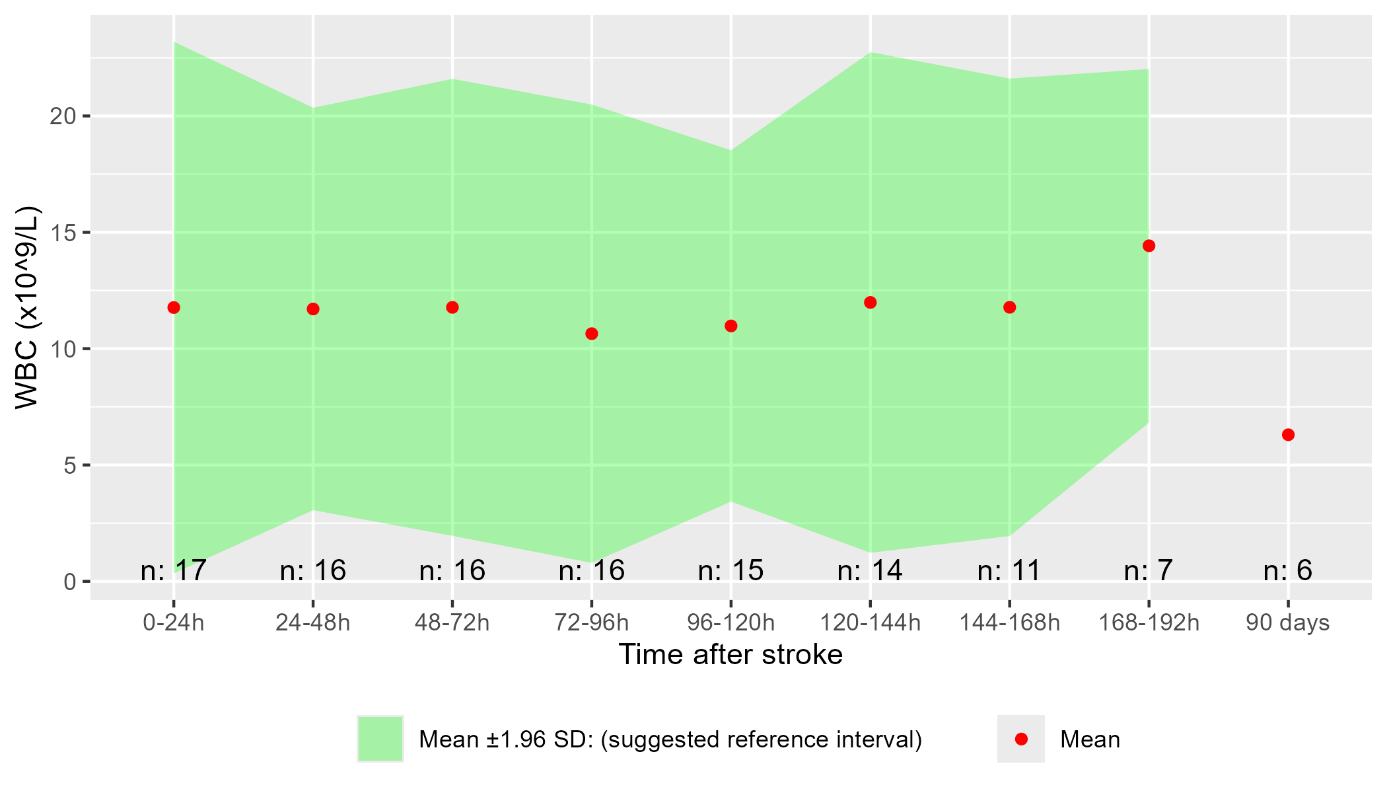


**Figure 17: WBC in patients with complications.** “n:” is the number of observations from the respective days. WBC: White Blood Cell count

## Body temperature

For body temperature, the mean temperature is about 0.5 degrees higher compared to patients without complications (Figure 18).


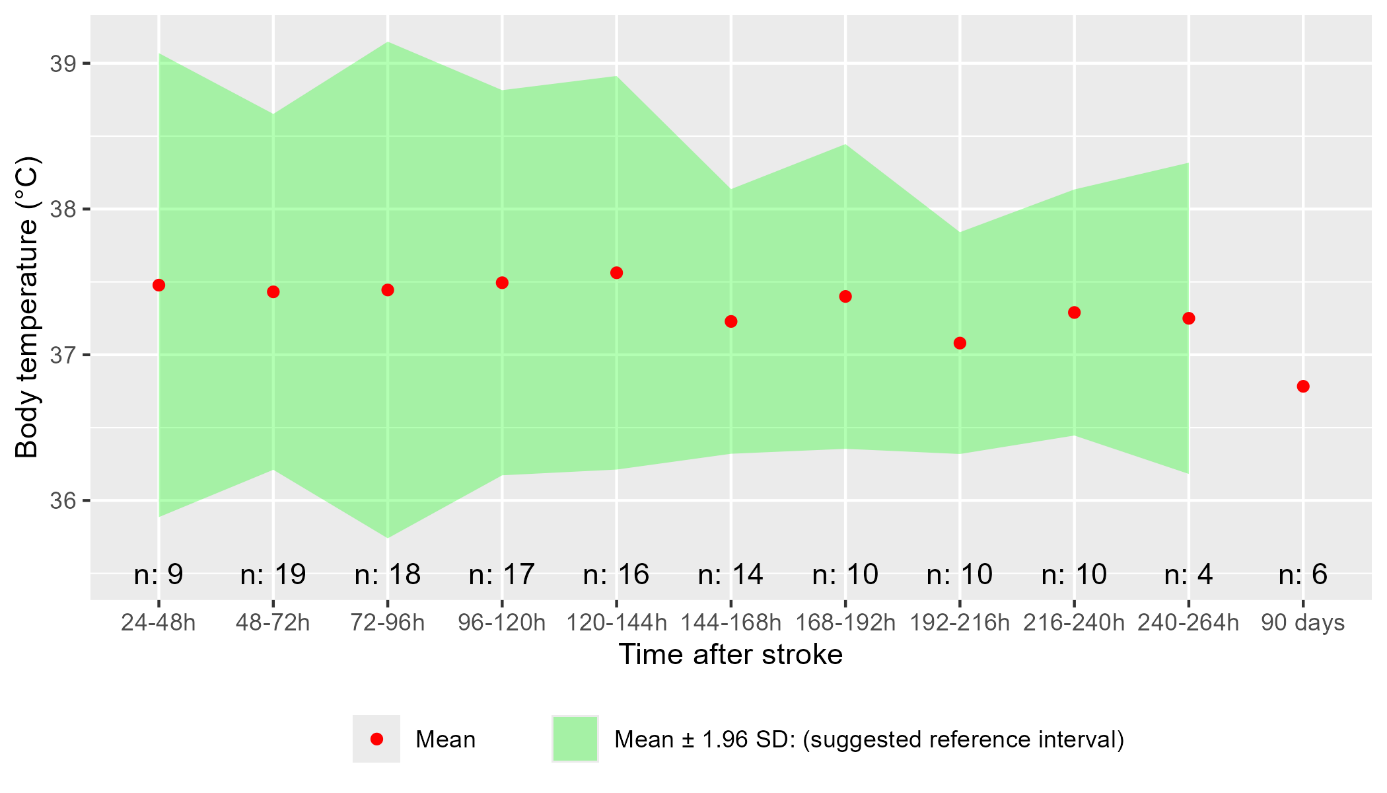


**Figure 18: Body temperature in patients with complications.** “n:” is the number of observations from the respective days.
